# Molecular Signatures of the Evolving Immune Response in Mice following a *Bordetella pertussis* Infection

**DOI:** 10.1371/journal.pone.0104548

**Published:** 2014-08-19

**Authors:** René H. M. Raeven, Jolanda Brummelman, Jeroen L. A. Pennings, Olaf E. M. Nijst, Betsy Kuipers, Laura E. R. Blok, Kina Helm, Elly van Riet, Wim Jiskoot, Cecile A. C. M. van Els, Wanda G. H. Han, Gideon F. A. Kersten, Bernard Metz

**Affiliations:** 1 Intravacc, Bilthoven, The Netherlands; 2 Division of Drug Delivery Technology, Leiden Academic Centre for Drug Research, Leiden, The Netherlands; 3 Centre for Infectious Disease Control, National Institute for Public Health and the Environment (RIVM), Bilthoven, The Netherlands; 4 Centre for Health Protection (GZB), National Institute for Public Health and the Environment (RIVM), Bilthoven, The Netherlands; University of Nebraska Medical Center, United States of America

## Abstract

Worldwide resurgence of pertussis necessitates the need for improvement of pertussis vaccines and vaccination strategies. Since natural infections induce a longer-lasting immunity than vaccinations, detailed knowledge of the immune responses following natural infection can provide important clues for such improvement. The purpose was to elucidate the kinetics of the protective immune response evolving after experimental *Bordetella pertussis* (*B. pertussis*) infection in mice. Data were collected from (i) individual analyses, i.e. microarray, flow cytometry, multiplex immunoassays, and bacterial clearance; (ii) twelve time points during the infection; and (iii) different tissues involved in the immune responses, i.e. lungs, spleen and blood. Combined data revealed detailed insight in molecular and cellular sequence of events connecting different phases (innate, bridging and adaptive) of the immune response following the infection. We detected a prolonged acute phase response, broad pathogen recognition, and early gene signatures of subsequent T-cell recruitment in the lungs. Activation of particular transcription factors and specific cell markers provided insight into the time course of the transition from innate towards adaptive immune responses, which resulted in a broad spectrum of systemic antibody subclasses and splenic Th1/Th17 memory cells against *B. pertussis*. In addition, signatures preceding the local generation of Th1 and Th17 cells as well as IgA in the lungs, considered key elements in protection against *B. pertussis*, were established. In conclusion, molecular and cellular immunological processes in response to live *B. pertussis* infection were unraveled, which may provide guidance in selecting new vaccine candidates that should evoke local and prolonged protective immune responses.

## Introduction

The gram-negative bacterium *Bordetella pertussis*, the causative agent of whooping cough (pertussis), accounted for high mortality rates among infants in the pre-vaccine era. Whole cell and acellular vaccines have drastically decreased the number of cases [1]. However, recently resurgence of pertussis in the vaccinated population has been observed in the USA [2], [3] and in European countries [4], [5]. Currently pertussis is still endemic, ranking in the top-ten of vaccine preventable diseases worldwide, according to the WHO. Next to improved diagnostics and public awareness, also strain adaptation and waning immunity are thought to be responsible for this increase in disease cases [6]. Thus, a call for vaccines with improved efficacy is evident.

As a main feature of the innate immune response upon *B. pertussis* infection in mice the recognition of lipopolysaccharide (LPS) by TLR4 has been acknowledged [7], [8]. This activation of TLR4 by *B. pertussis* leads to up-regulation of cytokine gene expression and recruitment of neutrophils into the lungs [9], [10]. It was found in animal models that *B. pertussis* infection results in formation of T-helper (Th) 1 and Th17 cells [11]–[13]. Since immunity induced by natural infections provides faster clearance upon reinfection and is longer lasting compared to both acellular and whole cell pertussis vaccination [14], [15], immune mechanisms induced upon infection or vaccination have been compared. In human and murine studies, immunization with whole cell or acellular pertussis vaccines results predominantly in a Th1 or a Th2 response, respectively [11], [16]. In addition, in both the intramuscular (human) or subcutaneous (mice) administered acellular and whole cell pertussis vaccines, the humoral response is characterized by systemic IgG [17], [18], while mucosal immune responses seem absent. Despite the absence of direct evidence for correlates of protection against *B. pertussis*, both Th1 and Th17 type CD4^+^ T-cells as well as IgA-producing B-cells seem to play an important role in a protective immune response against *B. pertussis* [12], [15], [19]–[22]. Since these responses are not induced by the currently available vaccines [23], but more resemble responses induced by infection, a better understanding of the immune response induced in the host following *B. pertussis* infection is needed. Despite knowledge on particular elements of the immune response generated by a *B. pertussis* infection, little is known about the kinetics and sequential relation of these elements. For this, systems biology can be an important tool, as was shown for tuberculosis and influenza infection [24]–[26].

Here, systems biology was applied to elucidate molecular and cellular events in the different phases of the immune response after primary *B. pertussis* infection in a murine model. To this end, innate and adaptive immune responses were investigated over a period of 66 days post infection. Gene expression profiles in spleen and lungs, cytokine profiles in sera, and cellular composition of the spleen were determined at twelve time points. Furthermore, cellular and antibody mediated immune responses against *B. pertussis* were investigated. Herewith, we revealed a chronological cascade of immunological processes consisting of recognition, processing, presentation and clearance of *B. pertussis*. The extensive insights into the immune response upon *B. pertussis* infection generated in this study may serve as a solid base for future research on pertussis vaccines and vaccination strategies.

## Results

### Lung clearance of infected mice

**Figure 1:**
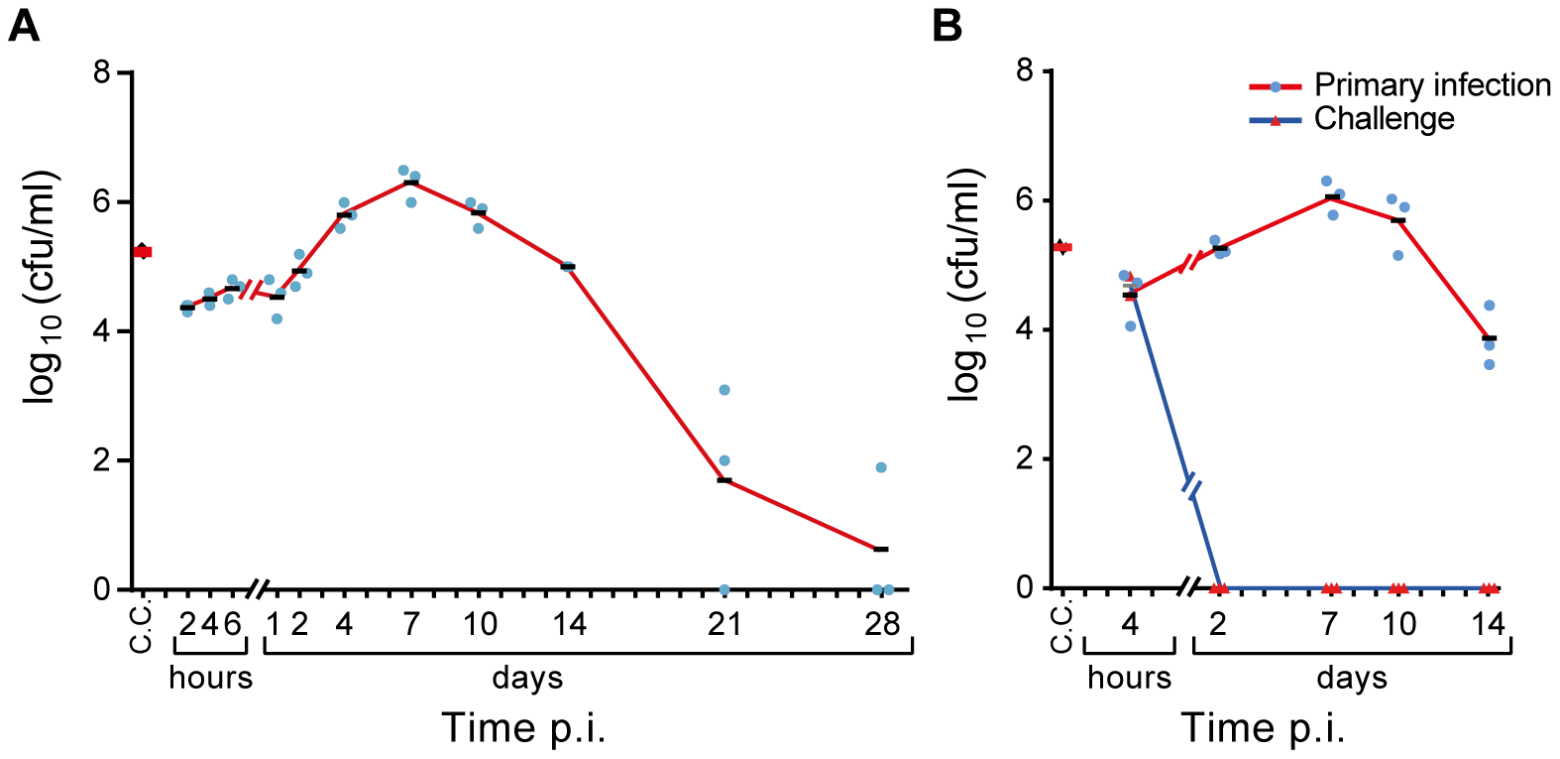
Lung clearance of naive and reinfected mice after *B. pertussis* infection. (A) Number of colony forming units (cfu) in challenge culture (C.C.) was confirmed before challenge. All other cfu were determined in lung of challenged mice (mean n = 3). A large fraction of the original infection dose was traceable in the lungs of mice 2 hours p.i. Bacteria were able to colonize and multiply approximately 100-fold at 7 days p.i. After one week, the mice were able to clear bacteria, which resulted in cleared lungs (2 out of 3 mice) at the last time point (day 28 p.i.). (B) Reinfection was performed at 56 days after primary infection and the number of cfu were counted after 4 hours p.i. While reinfected mice were able to clear *B. pertussis* from the lungs within 2 days p.i., naive mice showed a similar pattern as observed in Figure 1A.

The presence of *B. pertussis* in lungs of mice was examined during a period of 28 days post infection (p.i.), providing the benchmark for this study (Figure 1A). Therefore, mice were intranasally infected with *B. pertussis* using a dose of 10^5^ colony forming units (cfu). Approximately 13% of the intranasal dose was traceable in the lungs of mice 2 hours p.i. The number of bacteria remained fairly constant for one day, and increased from the second day to a maximum 7 days p.i. (10^7^ cfu). Subsequently, a decrease in the number of bacteria was observed and complete clearance in 2 out of 3 mice was achieved 28 days p.i. To determine whether single intranasal infection with *B. pertussis* leads to protection, mice were reinfected 56 days after primary infection (Figure 1B). A similar number of viable bacteria was detected 4 hours p.i. in lungs of both reinfected and naive mice. Reinfected mice were able to clear *B. pertussis* from the lungs within 2 days p.i., whereas naive mice showed a similar pattern as observed before. In conclusion, naive mice can clear *B. pertussis* from the lungs in about 28 days. Furthermore, mice previously infected with *B. pertussis* had developed sterilizing immunity, which clears the lungs in two days.

### Gene expression in lung tissue

**Figure 2:**
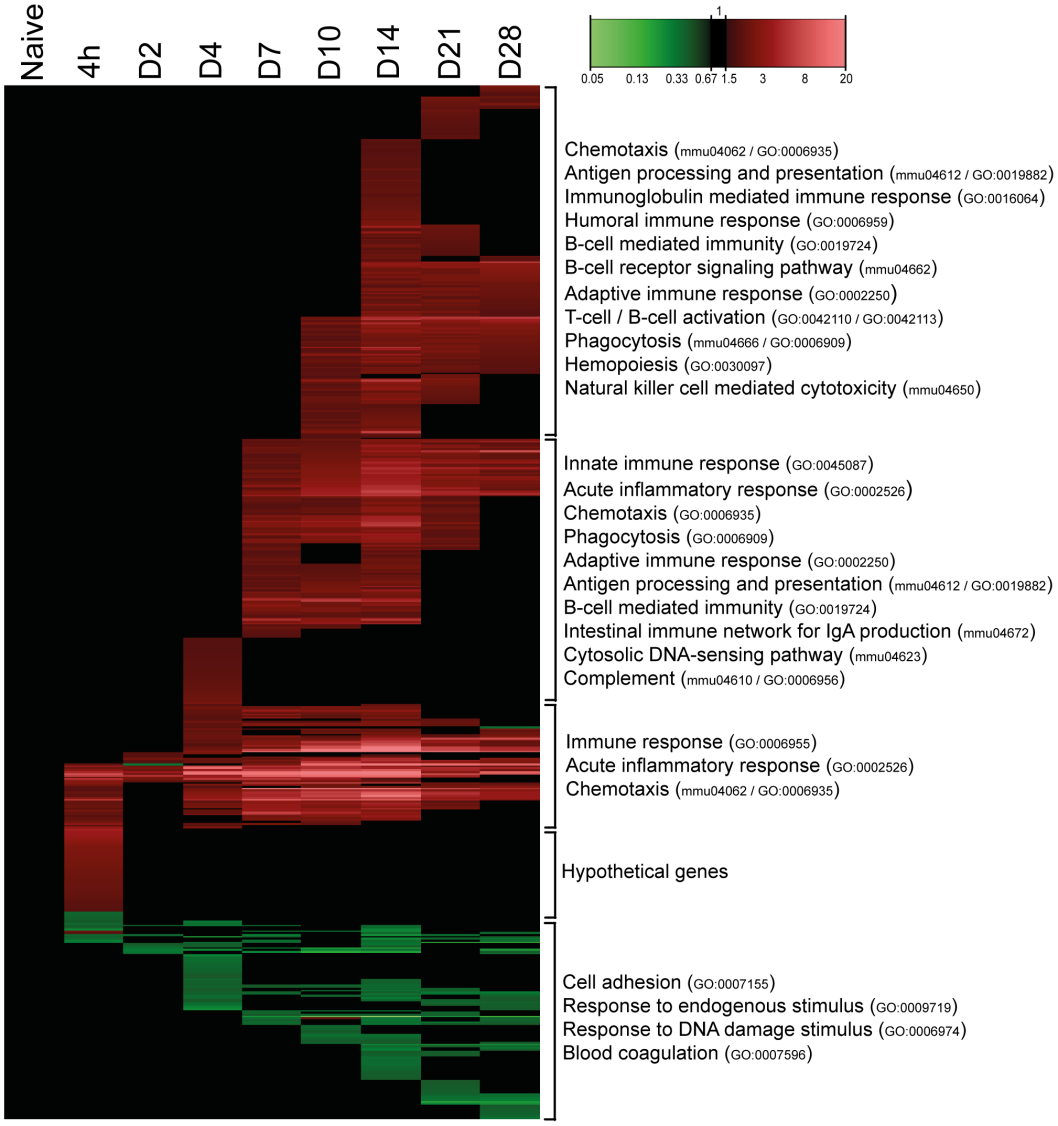
Pulmonary gene expression profiles as function of time after *B. pertussi*s infection. Fold changes in expression were calculated compared to naive mice and significant gene expression results (FR≥1.5, *p*-value≤0.001) are visualized as heatmap (mean of n = 3). In total, 558 genes were found to be differentially regulated, divided in 446 up-regulated genes (red) and 112 down-regulated genes (green). Genes not exceeding a fold change of 1.5 are depicted as basal level (black) at this time point. Results are divided in 5 clusters representing up/down-regulation and time of involvement including corresponding GO-BP terms and KEGG pathways.

**Figure 3:**
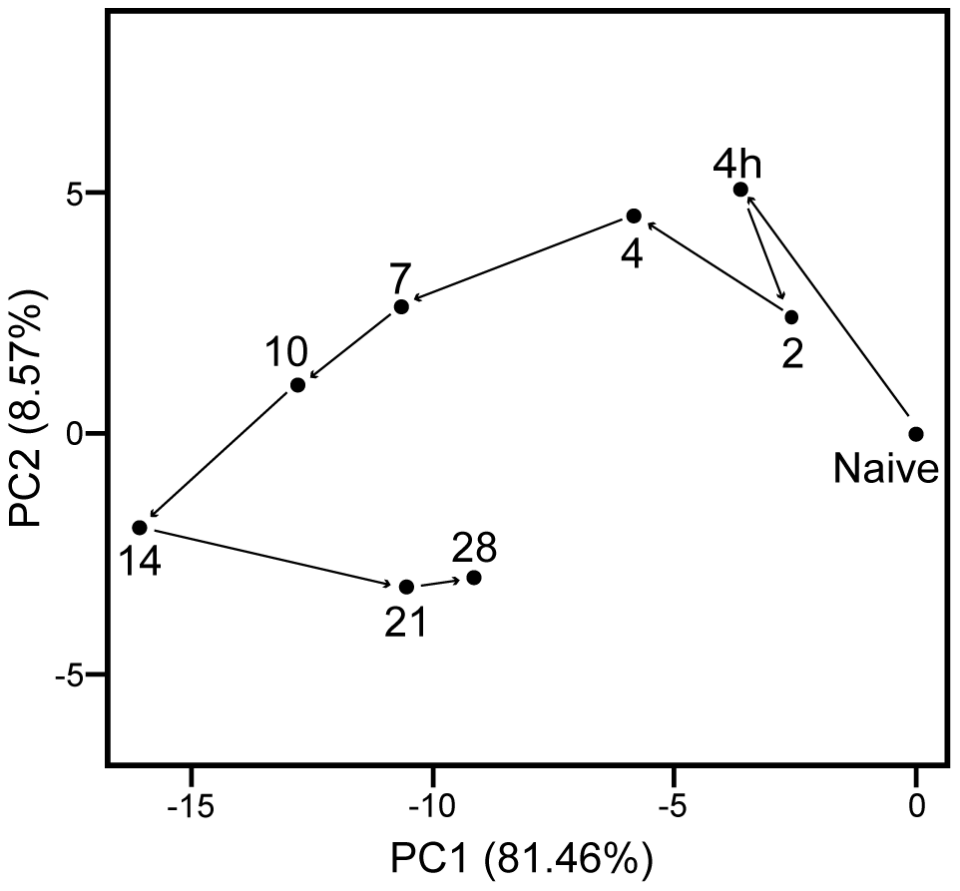
Principal component analysis of pulmonary gene expression. An evolving gene expression profile was indicated after *B. pertussis* infection of naive mice (Mean of n = 3), illustrated as principal-component analysis (PCA). PCA is a mathematical algorithm [117], which describes data based on (dis)similarity. Therefore, a greater distance between points in the plot corresponds to a greater dissimilarity. In this figure, the similarity of the 10 time points are compared based on the expression profiles of the 558 differentially expressed genes. Results indicate that 81% and 9% of the variance between time points could be addressed by principal component 1 (PC1) and PC2, respectively. The next principle components contribute for the residual variances. The plot revealed a notable change in gene expression at 2 days p.i. compared to 4 hours p.i., and largest differences at 14 days p.i. The gene expression at 28 days p.i. was still far from the naive state.

The gene expression in lung tissue of infected mice was monitored over a period of 28 days. In total 558 genes of the genome were differentially regulated (*p*-value≤0.001, fold ratio (FR) ≥1.5): 446 genes were up-regulated and 112 genes were down-regulated (Figure 2). A complete list of gene names is given in Table S1. Gene expression profiles changed already 4 hours p.i. with largest differences at 14 days p.i. (377 responsive genes). The number of genes with an altered expression had declined 21 and 28 days p.i. However, the expression of many genes had not yet returned to naive basal levels. Principal component analysis confirmed the largest differences at 14 days p.i. and the decline at 21 and 28 days p.i. (Figure 3). Interestingly, the time point with the least changes in gene expression (2 days p.i.) preceded the increase in cfu in the lungs (Figure 1).

**Figure 4:**
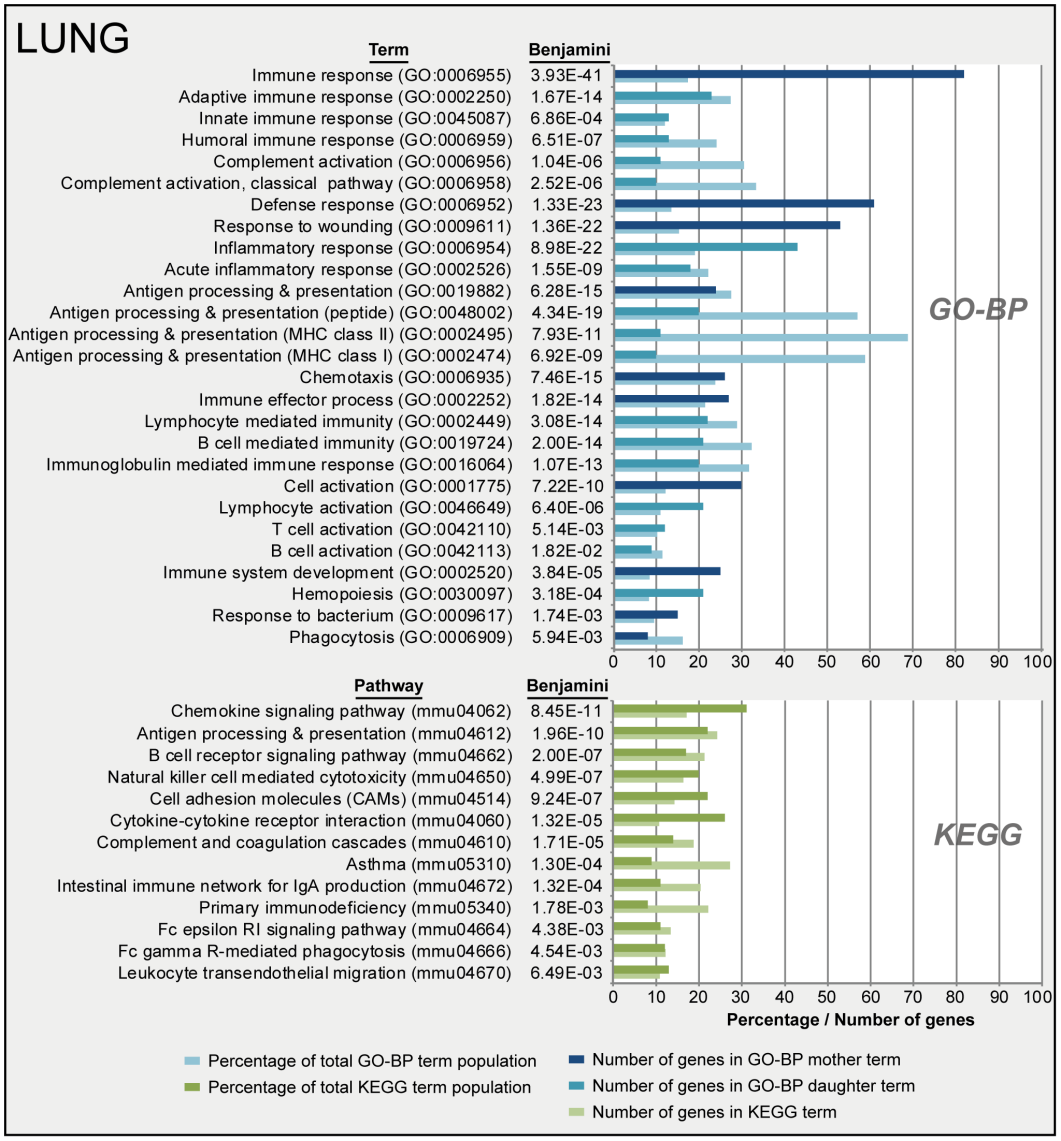
Functional annotation of pulmonary gene expression data after *B. pertussis* infection. Over-representation analysis (Benjamini-corrected *p*-value≤0.05) in GO-BP and KEGG databases of pulmonary gene expression data resulted in respectively 122 terms and 20 pathways significantly enriched. A selection of terms and pathways, based on immunological relevance and exclusion of overlapping terms, is given in this figure. GO-BP terms were divided in mother terms (dark blue) and corresponding daughter terms (light blue). For each term the amount of genes, the percentage of the total term population and the Benjamini-corrected *p*-value are shown.

To determine which biological processes were involved, functional analysis of all 558 genes was performed using DAVID. Over-representation analysis (ORA) (Benjamini-corrected *p*-value≤0.05) in GO-BP and KEGG databases resulted in respectively 122 terms and 20 pathways significantly enriched. A selection of these terms is presented in Figure 4. The selection is based on immunological relevance and by exclusion of overlapping terms. Acute phase response and chemotaxis were observed right after infection (4 hours p.i.) (Figure 2). Subsequently, innate immune response, phagocytosis, antigen processing and antigen presentation were initiated (7 days p.i.). Involvement of B-cell mediated immunity in this stage is mainly demonstrated by the expression of complement related genes. Eventually, the gene expression showed initiation of the adaptive immune response, such as activation of B-cells and T-cells, B-cell receptor (BCR) signaling, humoral immune response (complement activation) and immunoglobulin-mediated immunity from 10 until 28 days p.i.

The gene expression data was compared with the BioGPS database to identify the influx, presence or activation of particular immunological cells in the lungs (Figure S1). Sixty-one genes, which are predominantly expressed in macrophages, suggest two events: (i) triggering of alveolar macrophages 4 hours p.i. and (ii) recruitment of macrophages in the lungs 7 days p.i. The influx of macrophages was observed on cellular level using fluorescence microscopy [27]. In addition, the increased expression of 29 genes was attributed to the presence of neutrophils in the lungs 4 days p.i. Moreover, data suggests altered expression of 32, 48 and 17 genes of B-cells, dendritic cells (DCs) and mast cells, respectively, 7 days p.i., and 19 T-cell genes 14 days p.i.

Differentially regulated genes in lung tissue after the infection with *B. pertussis* were classified according to function, pathway and cell type. For ten different groups details are described below.

#### Cytokines

**Figure 5:**
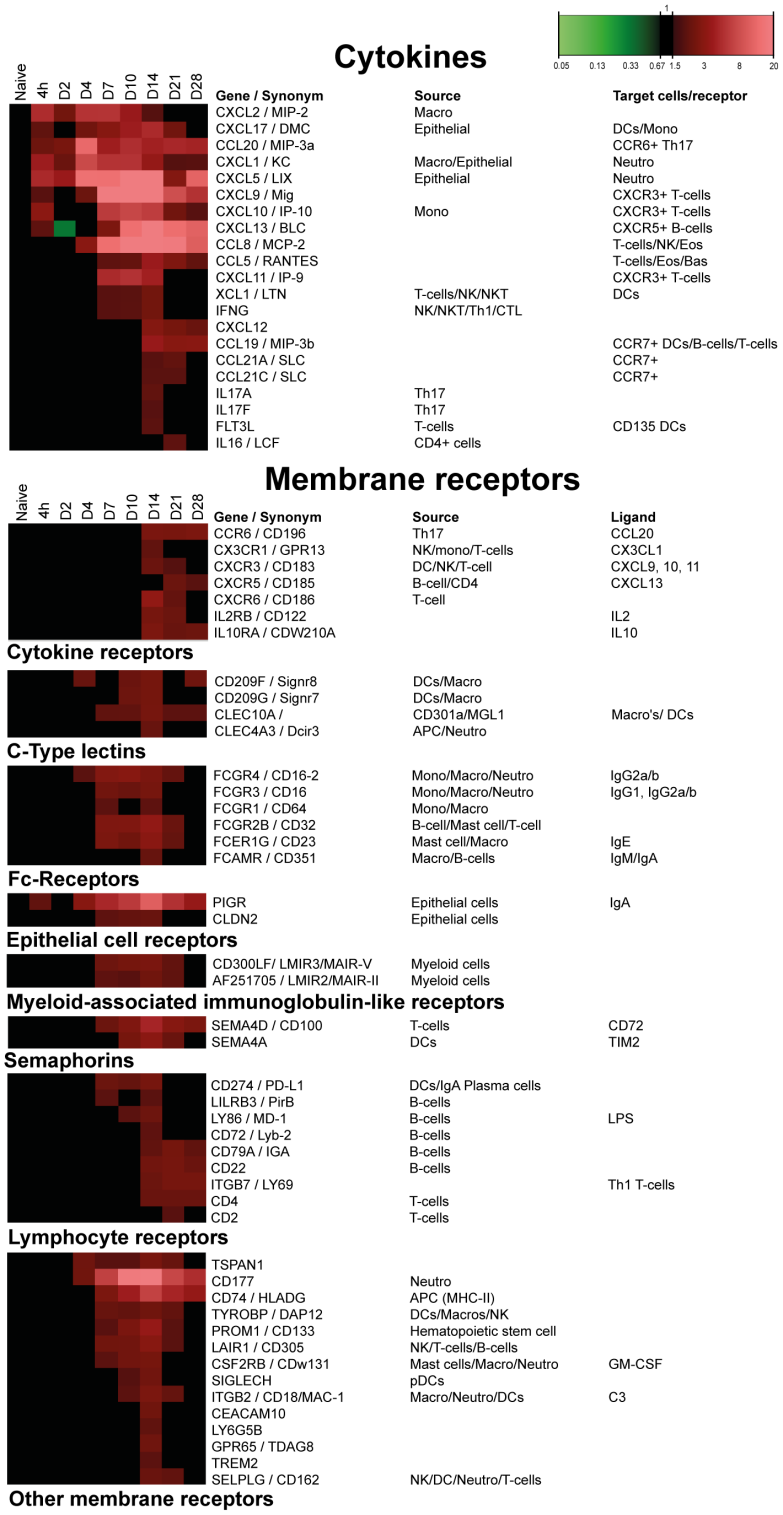
Gene expression profiles of pulmonary cytokines and membrane receptors after *B. pertussis* infection. Results for cytokines and membrane receptors include expected source and known corresponding receptors, target cells and receptor ligands. Membrane receptors are divided in eight different groups according to function. (Mean of n = 3).

Twenty-one cytokine-encoding genes were differentially expressed in the lungs during *B. pertussis* infection (Figure 5). Sixteen cytokines are associated with a chemotactic function. The other five genes (*Flt3l*, *Ifng*, *Il17a*, *Il17f*, *Il16*) reflect cytokines involved in the activation of T-cells, such as Th1 and Th17 cells.

#### Membrane receptors

Forty-six membrane receptors were identified with altered gene expression (Figure 5). The receptors were divided in eight groups: cytokine receptors, C-type lectins (CLRs) [28], Fc-receptors, epithelial cell specific receptors, including the IgA transporter (pIgR), myeloid-associated immunoglobulin-like receptors, semaphorins [29], lymphocyte receptors (e.g., *Pdl1*) [30], [31] and the mucosal-homing receptor (*Itgb7*). Other membrane receptors are collected in one group (Figure 5).

Gene expression profiles of both cytokines and cytokine receptors enabled early prediction of immune cell recruitment towards the lungs on transcriptomic level in this study. Increased expression of *Cxcl13* and *Ccl20* was followed by the up-regulation of receptors *Cxcr5* and *Ccr6*. These chemokines lead to the recruitment of CXCR5+ B-cells, CXCR5+ T-cells, CCR6+ DCs and CCR6+ Th17 cells into the lungs [32]–[34]. Likewise, the expression of *Cxcl9*, *Cxcl10* and *Cxcl11*, induced by interferon gamma (IFNγ), is associated with recruitment of CXCR3+ T-cells or CXCR3+ pDCs [35].

#### Antimicrobial peptides (AMPs)

**Figure 6:**
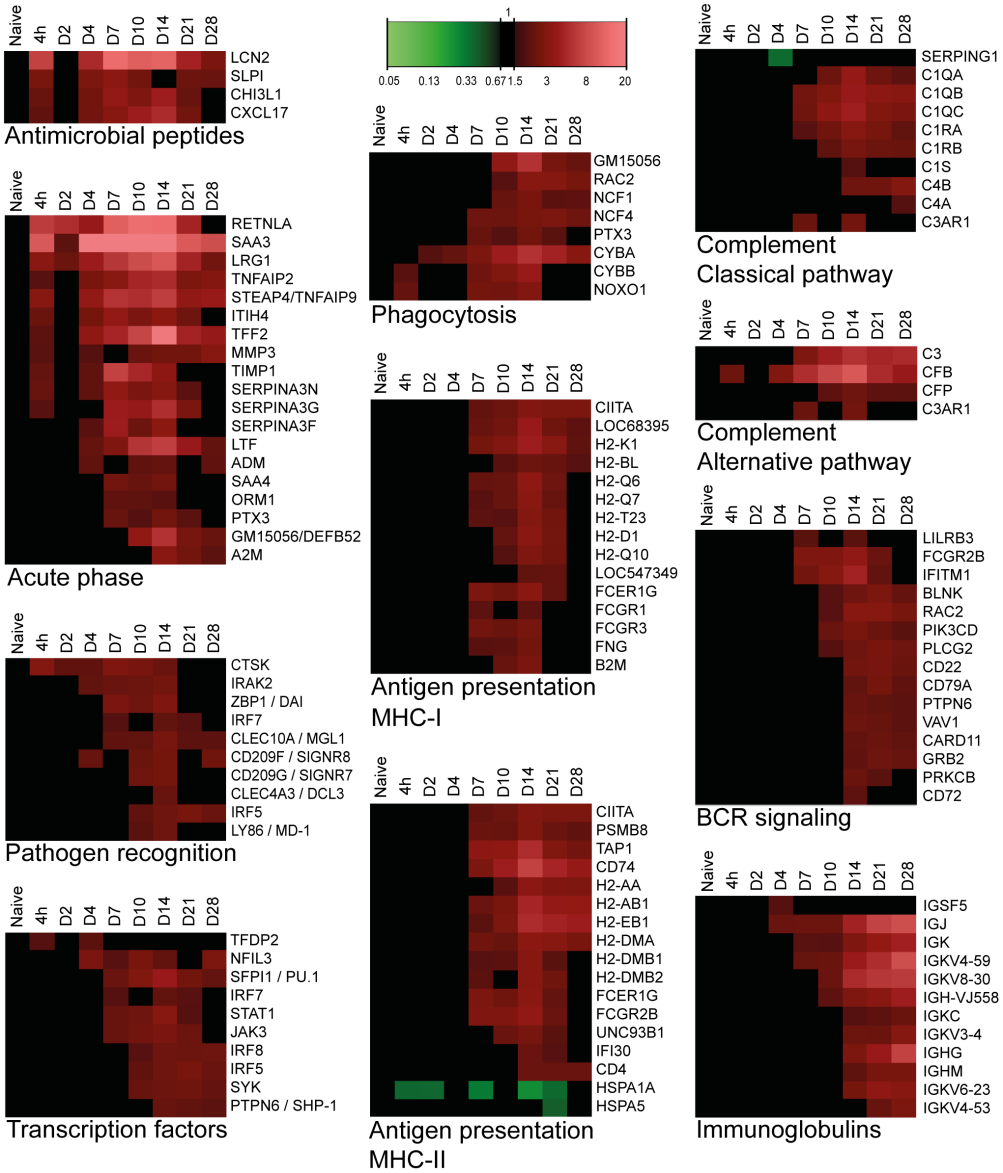
Pulmonary gene expression representing immunological pathways as function of time after *B. pertussis* infection. Details on pulmonary gene expression profiles indicated expression of anti-microbial peptides, acute phase proteins, pathogen recognition, phagocytosis, both classical and alternative pathways of complement cascade, transcription factors, antigen presentation via both MHC-I and MHC-II, B-cell receptor (BCR) signaling and immunoglobulin related genes. (Mean of n = 3).

AMPs have defensive properties against bacterial infections, recruit immune cells and model subsequent immune responses [36]. Gene expression of four AMPs was elevated for 21 days p.i. (Figure 6). These four AMPs recruit neutrophils or protect against respiratory pathogens [37]–[40]. Interestingly, the chemokine *Cxcl17*, primarily expressed in the lungs, was described as an antimicrobial mucosal chemokine that recruits DCs and CD14^+^ monocytes and protects against pathogenic bacteria [41], [42].

#### Acute phase response

Primary reaction to an infection is the acute phase response, which leads to the release of pro-inflammatory cytokines and activation of inflammatory cells. Strong up-regulation of 19 genes involved in the acute phase response was detected at 4 hours p.i. Remarkably, the gene expression of the acute phase response remained highly expressed at least until day 28 p.i. (Figure 6).

#### Pathogen recognition

Enhanced gene expression suggested activation of TLR4, TLR9, DAI and CLR signaling pathways, implying pathogen recognition by several ligands of *B. pertussis* (Figure 6). The induction of *Md-1* and transcription factor *Irf5* genes suggested TLR4-signaling upon sensing of LPS [43]. Up-regulation of *Ctsk* and the downstream transcription factor *Irf7* assumes activation of TLR9 by sensing of unmethylated CpG sequences in bacterial DNA [44]. In addition, the cytosolic DNA sensor (DAI) was induced [45]. Elevated gene expression of several CLRs was noticed, such as Mgl1 and the mouse DC-SIGN-related proteins *Signr7* and *Signr8* [46]. Mgl1 recognizes *N*-acetylgalactosamine (GalNAc), which is present in *B. pertussis*. Mgl1 has recently been linked to *B. pertussis* mast cell interaction [47].

#### Transcription factors

Ten transcription factors were differentially expressed in the lungs (Figure 6). These include genes of factors *Pu.1*, *Shp-1*, *Nfil3*, *Syk*, *Irf5*, *Irf7* and *Irf8*. All of these transcription factors have been designated as regulators of immunological processes, thereby defining the direction of the host's immune response upon bacterial infection [48]–[54].

#### Phagocytosis

Six genes representing subunits of NADPH oxidase (NOX) were activated between 4 hours and 28 days p.i. (Figure 6). The activation suggests the production of reactive oxygen species, which are involved in a phagocyte respiratory burst to assist in the killing of bacteria during phagocytosis [55].

#### Antigen presentation

Twenty-eight genes representing antigen presentation were up-regulated between 7 and 28 days p.i. (Figure 6). Results indicate that both antigen presentation via MHC class I and class II were included. This suggests antigen presentation to both CD4^+^ T-helper cells and cytotoxic CD8^+^ T-cells (CTLs). In line with these findings, the up-regulation of *Cd4* and *Ctsw gene expression* in lung tissue was observed, which may indicate presence of Th-cells [56] and CTLs [57], respectively (Figure S1).

#### Complement activation

Twelve genes of the complement system were more abundantly expressed between 7 and 28 days p.i. (Figure 6). Increased gene expression was observed for the complement components C1 and C4, which belong to the classical pathway. In contrast, reduced gene expression of the C1-inhibitor (*Serping1*) was observed day 4 p.i. Furthermore, strong up-regulation of *Cfb* suggests activation of the alternative complement pathway.

#### B-cell receptor (BCR) signaling

Fifteen genes of the BCR signaling pathway were up-regulated between 7 and 28 days p.i. (Figure 6). BCR activation was detected by elevated gene expression of eight downstream proteins (*Blnk*, *Card11*, *Grb2*, *Pik3cd*, *Plcg2*, *Prkcb*, *Rac2* and *Vav1*). Furthermore, the gene expression of the BCR signaling pathways indicates a balanced B-cell response because of an interplay of stimulation by *Cd79a* and *Ifitm1*, and inhibition by *FcgrIIb*, *Lilrb3* (*Pirb*), *Cd22*, *Cd72* and *Shp-1*. These co-inhibitors prevent overstimulation of B-cells [58].

#### Immunoglobulins

Gene expression associated with antibody formation was observed after 4 days p.i. and was increased up to 28 days p.i. (Figure 6). The microarray data revealed gene expression of seven kappa (κ) light chain genes, heavy chain Ig alpha chain C (*Ihg-vj558*), *Ighm* and *Ighg*, indicating the formation of IgA, IgM and IgG.

In summary, approximately 40% of the genes expressed in the lungs could be annotated according to function. The chronology of immunological processes comprises broad pathogen recognition, prediction of cell recruitment by cytokine and receptor expression, MHC class I and II antigen presentation, and markers for both antibody and cell-mediated immunity.

### Cytokine profiles in mouse sera

**Figure 7:**
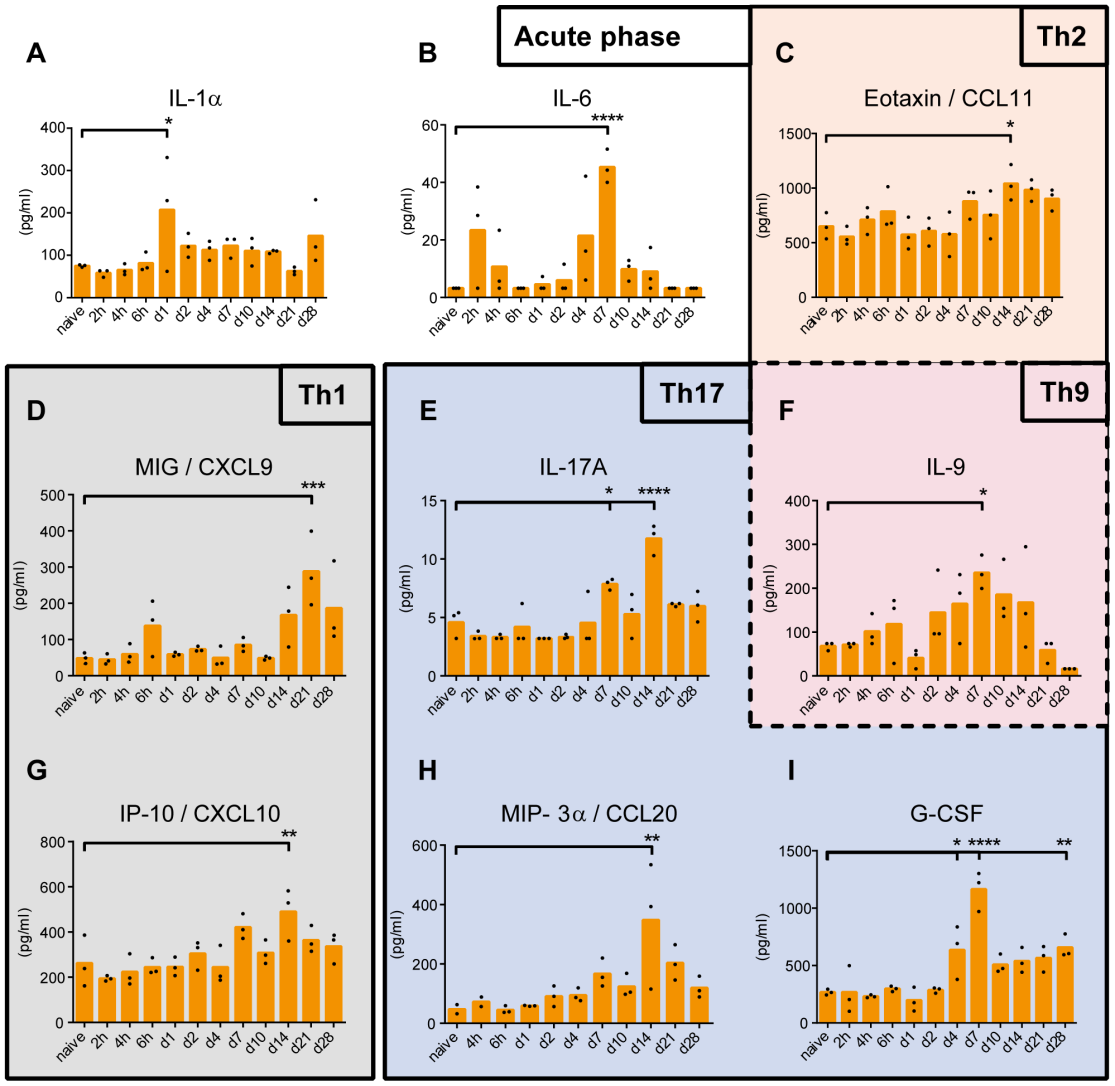
Serum cytokine concentrations as function of time after *B. pertussis* infection. The concentrations of (A) IL-1α, (B) IL-6, (C) CCL11, (D) CXCL9, (E) IL-17A, (F) IL-9, (G) CXCL10, (H) CCL20, and (I) G-CSF in serum were analyzed over time. Results indicated that after the acute phase response (IL-1α and IL-6) a multi-flavored immune response with Th1 (CXCL9 and CXCL10), Th2 (CCL11 and IL-9) and Th17 (G-CSF, CCL20, IL-9 and IL-17A) was induced. Data represented as mean concentrations including individual values (n = 3). *p*-values were determined by one-way ANOVA with multiple comparison compared to naive mice (* = *p*<0.05, ** = *p*<0.01, *** = *p*<0.001 and **** = *p*<0.0001).

The concentration of thirty-three cytokines was determined in mouse sera by ELISA and multiplex immunoassay (MIA). *B. pertussis* infection induced nine cytokines: CCL11, CCL20, CXCL9, CXCL10, G-CSF, IL-1α, IL-6, IL-9 and IL-17A (Figure 7). The pro-inflammatory cytokines, IL-1α and IL-6, are both involved in the acute phase response. The concentration of IL-1α was significantly increased 1 day p.i. Enhanced concentrations of IL-6 were detected at three different time points: 2 hours (not significant), 4 days, and 7 days p.i. (Figure 7A). Remarkably, IL-6 concentrations returned to basal level 6 hours p.i. Production of IL-1α by epithelial cells was most likely a result of LPS or tracheal cytotoxin (TCT) exposure [59]. The enhanced concentration of IL-6 2 hours p.i. was most likely caused by lung-resident immune cells, such as macrophages and mast cells. These cells are the firstto recognize bacterial infection [13]. Elevated concentrations of chemokines CXCL9 (MIG) and CXCL10 (IP-10) were measured between 14–28 days p.i., which suggests a Th1 response [60]. CCL11 (Eotaxin) was enhanced at 14 days p.i. (Figure 7E). Eotaxin is involved in allergic inflammation and is chemoattractive for eosinophils [61] and CCR3^+^ Th2 lymphocytes [62]. IL-9 was increased 7 days p.i. (Figure 7F). IL-9 is mainly produced by T-cells and originally associated with Th2 responses [63]. However, there is evidence that IL-9 is produced by a specific T-cell subset: Th9 cells [64] and Th17 cells [65]. A Th17 response was identified by markers, such as IL-17A, CCL20 (MIP-3α) and granulocyte colony-stimulating factor (G-CSF) [34]. Enhanced concentrations of IL-17A and CCL20 were determined in mouse sera 14 days p.i. (Figure 7G–H). CCL20 binds exclusively to CCR6, which is highly expressed on Th17 cells [32], [33]. G-CSF was produced between 4 and 28 days p.i. G-CSF is important for recruitment, differentiation and proliferation of neutrophils [66] and is linked to a Th17 response [67]. No significant changes in concentrations were detected for the other twenty-four cytokines.

In conclusion, serum cytokine profiles indicate the induction of an acute phase response (IL-1α and IL-6) followed by a multi-flavored immune response with Th1 (CXCL9 and CXCL10), Th2 (CCL11 and IL-9) and Th17 signatures (G-CSF, CCL20, IL-9 and IL-17A).

### Cellular composition of the spleen

**Figure 8:**
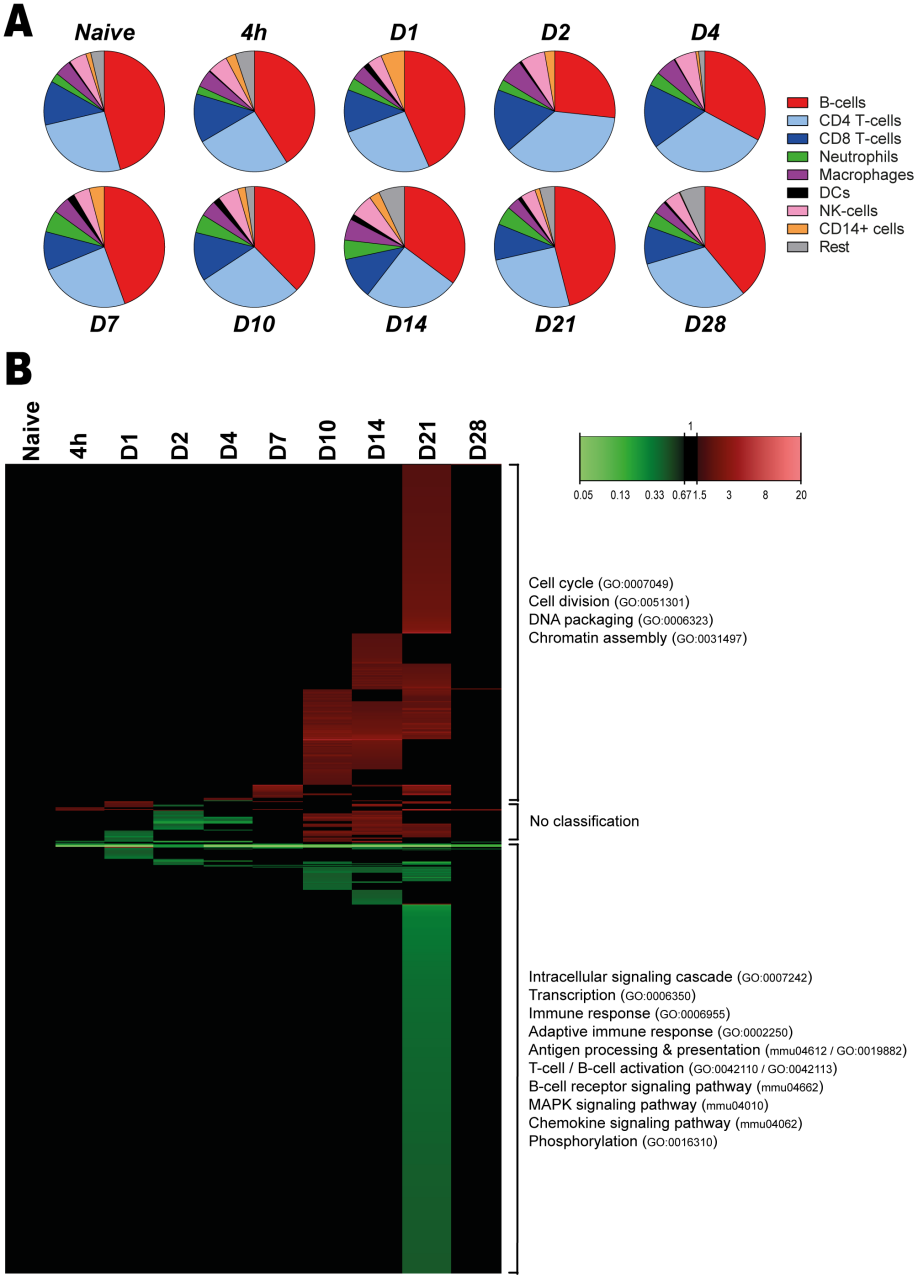
Splenic cellular composition and gene expression profiles as function of time after *B. pertussis* infection. (A) The distribution of the splenic cellular composition illustrated as pie charts at a selection of time points during the *B. pertussis* infection. FACS was used to determine the percentage (mean of n = 3) of B-cells (CD19^+^), CD4^+^ T-cells, CD8^+^ T-cells, neutrophils (Gr-1^+^), macrophages (CD11b^+^), dendritic cells (33D1^+^), NK-cells (DX5^+^) and CD14^+^ cells (CD14^+^) in splenocytes. (B) Fold changes of expression were calculated compared to naive mice and significant gene expression results (FR≥1.5, *P*-value≤0.001) were visualized as a heatmap (Mean of n = 3). This test was performed for each gene at each time point. In total, 798 genes were differentially regulated (FR≥1.5, *p*-value≤0.001) compared to naive mice, divided in 342 up-regulated (red) and 425 down-regulated genes (green). Additionally, a distinct group of 31 genes showed down-regulation between 4 hours and 4 days p.i., but were up-regulated between 10 and 21 days p.i. Genes not exceeding a fold change of 1.5 are depicted as basal level (black) at this time point. Results are divided in 3 clusters representing up/down-regulation and time of involvement and included corresponding GO-BP terms and KEGG pathways.

The cellular composition of the spleen was examined (Figure 8A and Figure S2) by flowcytometry with a panel of fluorescence-labeled monoclonal antibodies binding to cell-specific membrane markers (Figure S3 and S4). Results indicate that in the naive situation, the majority of splenocytes consist of B-cells and T-cells (approximately 40% each). Upon *B. pertussis* infection, the ratio between B-cells and T-cells changed, since an increased percentage of T-cells and decreased percentage of B-cells was observed on day 2–4 p.i. However, this ratio was restored on day 7 p.i. The distribution of minor cell populations in the spleen, such as DCs, neutrophils and CD14^+^ cells, changed over time upon *B. pertussis* infection. Here, we observed an early increased percentage of CD14^+^ cells (day 1 p.i.) and DCs (day 1 and day 7–14 p.i.). Furthermore, a gradual increased percentage of neutrophils from day 4 until day 21 p.i. was observed. Importantly, enlargement of the spleen was observed during the course of infection (not quantified), suggesting influx or proliferation of cells. This might explain the observed changes in cellular distribution.

In conclusion, cellular composition of the spleen indicates fluctuations over time upon *B. pertussis* infection.

### Gene expression in the spleen

**Figure 9:**
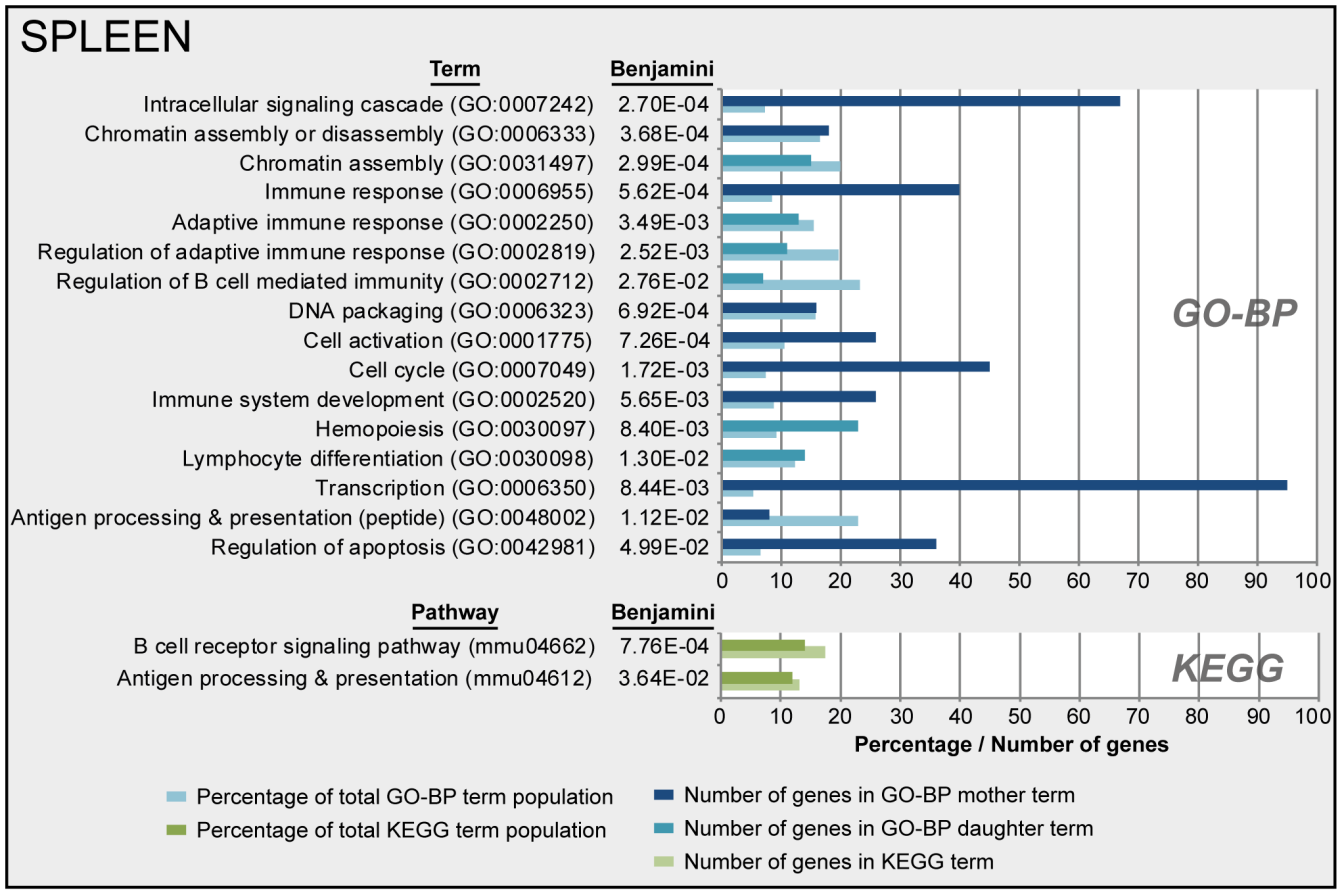
Functional annotation of splenic gene expression data after *B. pertussis* infection. Over-representation analysis (Benjamini-corrected *p*-value≤0.05) of all 798 differentially expressed genes in the spleen in GO-BP and KEGG databases, This resulted in respectively 66 terms and 2 pathways significantly enriched of which a selection, based on immunological relevance and exclusion of overlapping terms, of 16 terms and 2 pathways are presented. GO-BP terms were divided in mother terms (dark blue) and corresponding daughter terms (light blue). For each term the amount of genes, the percentage of the total term population and the Benjamini-corrected *p*-value are shown.

**Figure 10:**
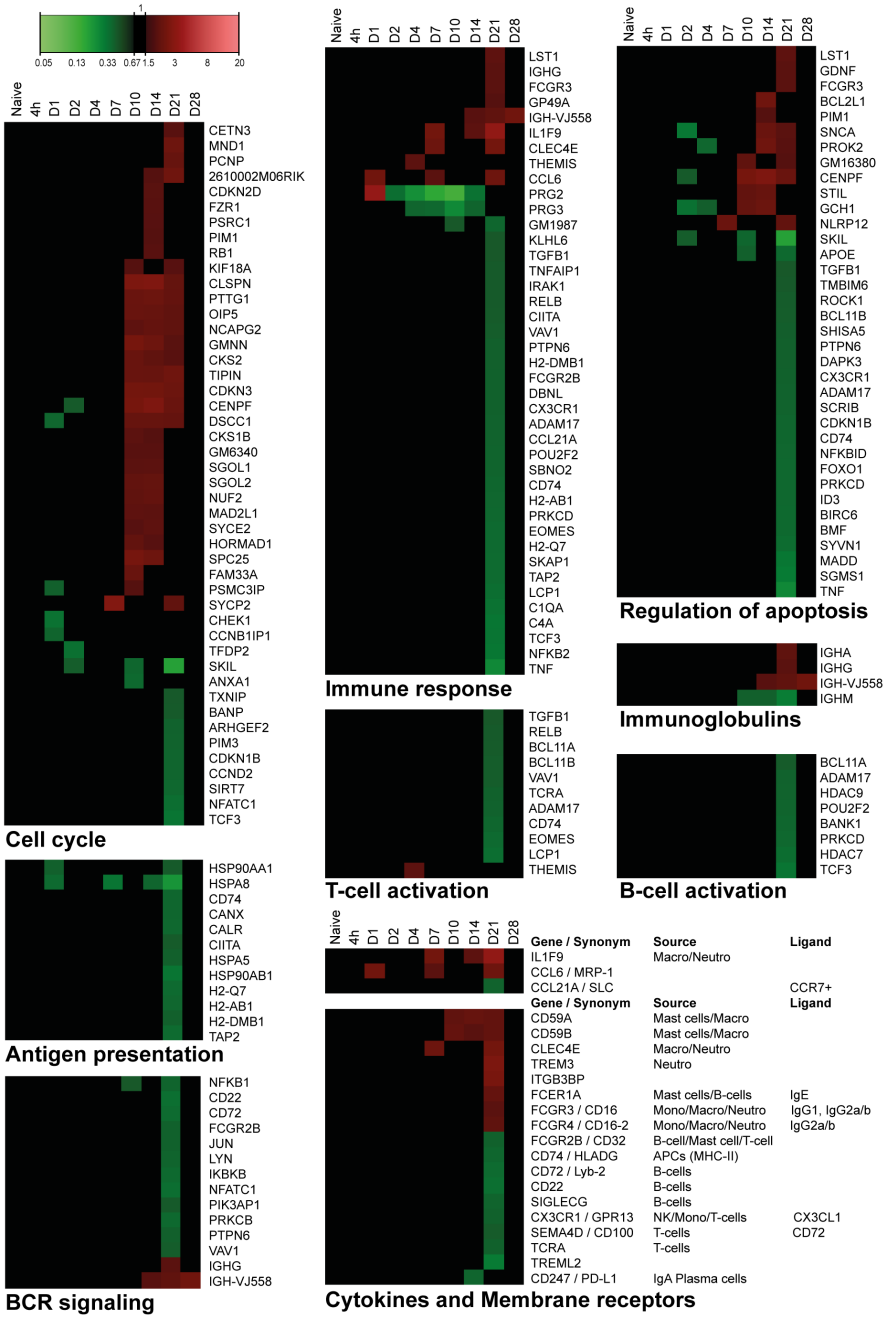
Splenic gene expression profiles of specific processes as function of time after *B. pertussis* infection. Expression profiles are summarized for immune response, cell cycle, regulation of apoptosis, T-cell activation, B-cell activation, immunoglobulin, B-cell receptor (BCR) signaling, antigen processing, cytokines and membrane receptors in spleen. (Mean of n = 3).

Gene expression in the spleen of infected mice was determined by microarray analysis. The study identified 798 genes of the whole genome that were differentially regulated (*p*-value≤0.001, FR≥1.5): 342 genes were up-regulated, 425 genes were down-regulated, and 31 genes that were initially down-regulated and subsequently up-regulated (Figure 8B). All involved genes are listed in Table S2. A limited set of genes showed altered gene expression between 4 hours and 7 days p.i. However, large changes in gene expression were observed 21 days p.i., while the gene expression was almost back to the original values 28 days p.i. Only eight genes still showed altered expression. Over-representation analysis (ORA) of the 798 differentially expressed genes in the spleen revealed 66 GO-BP terms and two KEGG pathways. A selection of 16 terms and 2 pathways is presented in Figure 9, which are involved in transcription, cell cycle, chromatin assembly and a number of immunological processes. The corresponding proteins are involved in proliferation, differentiation or activation of immune cells in the spleen. Down-regulated genes encode proteins that are involved in transcription, adaptive immune responses, antigen processing, antigen presentation and chemokine signaling (Figure 8B and 10). Down-regulation of these genes suggests suppression of the immune response or migration of immune cells.

#### Immune response

Forty-one immune response genes were identified (Figure 10), 13 of which are associated with the adaptive immune response. Interestingly, down-regulation of genes involved in T-cell activation, B-cell activation, antigen processing and presentation, and the subsequent BCR signaling pathway were observed 21 days p.i. (Figure 10). Up-regulation of genes encoding immunoglobulin heavy chains *Ighg*, *Igha* and *Igh-vj558* indicate production of immunoglobulins IgG and IgA by B-cells (Figure 10). Down-regulation of the *Ighm* gene suggests a suppressed IgM production or a class-switch to IgG or IgA.

#### Membrane receptors

Up-regulation of genes encoding myeloid cell membrane markers, Fc-receptors and membrane attack complex (MAC) inhibitory proteins (*Cd59a*/*Cd59b*) suggests increased presence of myeloid cells, such as neutrophils, between 10 and 21 days p.i. (Figure 10). The induction of the MAC-inhibitory genes suggests inhibition of MAC formation, which is part of the complement cascade. In addition, BioGPS analysis (Figure S5) revealed the presence of myeloid cell markers that are expressed on neutrophils (*Trem3* and *Clec4e*) or macrophages (*Clec4e*). Down-regulated gene expression of membrane markers of B-cells (*Cd22*, *Cd72*, *Cd74* and *Siglecg*) and T-cells (*Sema4d*, *Tcra*) implies decreased presence of lymphocytes 21 days p.i. (Figure 10).

#### Transcription

Ninety-five genes that were differentially regulated in the spleen turned out to be involved in transcription (Figure 9), of which 70 genes were down-regulated (data not shown).

#### Cell cycle

Most of the genes involved in the cell cycle process (31 out of 45) were up-regulated (Figure 10) which suggest increased cell replication between 10 and 21 days p.i. in the spleen.

#### Regulation of apoptosis

Apoptosis was characterized by up-regulation of 12 genes (between 7 and 21 days p.i.) and down regulation of 24 genes (21 days p.i.) (Figure 10). The role of apoptosis is especially interesting because of the recently described link between apoptotic markers and influenza vaccine responsiveness [68]. However, no overlap was found between the markers from the study of Furman *et al.* and the current study.

In summary, most prominent differences in gene expression in the spleen were found around 21 days p.i. These differences point to down-regulation of immunological processes. After 28 days p.i. the expression profiles returned to basal level, at the same moment that the bacteria were almost cleared from the lungs. Interestingly, when combining cellular composition (Figure 8A) with gene expression profiles (Figure 8B and Figure S5), the increase in neutrophils (10–21 days p.i.) was observed with both techniques. However, changes in the distribution of B-cells and T-cells on day 2 and 4 p.i. were not detected on gene expression level. Overall, these findings suggest that gene expression profiles cannot directly be translated in cellular fluctuations in the spleen.

### Overlap of transcriptomic responses in lungs and spleen

A comparison of transcriptomic datasets was made to determine the related biological processes that occurred in both spleen and lungs (Figure S6). Seventy-two genes were differentially expressed in spleen and lungs, which were divided in four groups: (i) 20 genes that were up-regulated in both tissues, (ii) 42 genes that were up-regulated in lungs and down-regulated in the spleen, (iii) 2 genes that were down-regulated in lungs and up-regulated in the spleen, and (iv) 8 genes that were down-regulated in both tissues. Fifteen genes of the second group were involved in the immune response, such as leukocyte activation, BCR signaling pathway, antigen processing and presentation, and chemotaxis. Remarkably, the immunoglobulin-mediated immune response was the only process up-regulated in both tissues, suggesting local and systemic antibody production.

In summary, both tissues showed activation of antibody-mediated immunity upon a *B. pertussis* infection. In addition, most differentially expressed genes representing immunological processes were observed up-regulated in the lungs and down-regulated in the spleen.

### Pertussis-specific IgM, IgG and IgA antibody responses

**Figure 11:**
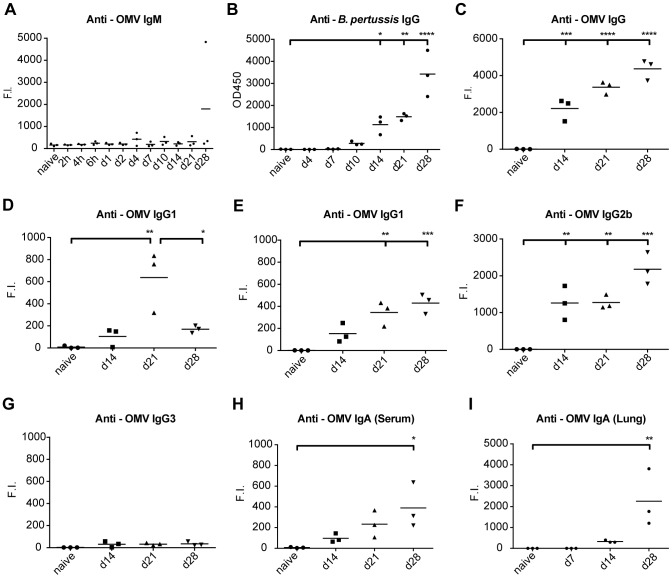
Antibody profiling after *B. pertussis* infection. Antibody responses were determined in mouse sera after intranasal infection by whole cell *B. pertussis* ELISA or MIA and expressed as OD450 or Fluorescent Intensity (F.I.) respectively. (A) IgM against OMV B1917 were absent. (B) Whole cell *B. pertussis* ELISA indicated IgG antibody formation 10–14 days p.i., which increased until day 28 days p.i. (C–G) Levels of total IgG and individual subtypes (IgG1, IgG2a, IgG2b and IgG3) against OMV B1917 indicated presence of multiple subclasses. (H) Anti-OMV IgA antibodies in serum were induced after 14–28 days p.i. (I) In lung lysates, anti-OMV IgA antibodies were detected 14 and 28 days p.i. I *p*-values were determined by one-way ANOVA with multiple comparison compared to naive mice (* = *p*<0.05, ** = *p*<0.01, *** = *p*<0.001 and **** = *p*<0.0001). (Mean of n = 3).

Antibody isotypes, IgM, IgG and IgA against *B. pertussis* were analyzed for a period of 28 days p.i. The formation of IgM against *B. pertussis* antigens present in outer membrane vesicles (OMV) was not observed during the course of infection (Figure 11A). Contrarily, anti-*B. pertussis* IgG in mouse sera was detectable from 10 days p.i. and the responses were progressively increased until 28 days p.i. (Figure 11B). Notably, no IgG titers were detectable against the individual purified *B. pertussis* antigens pertactin (Prn), pertussis toxin (Ptx), filamentous hemagglutinin (FHA) or fimbria type 2 and 3 (Fim2/3) (Figure S7). However, anti-OMV IgG was significantly increased at 14, 21 and 28 days p.i. (Figure 11C) and consisted of IgG1, IgG2a and IgG2b subclasses, but not IgG3. (Figure 11D–G), Remarkably, IgG1 levels dropped significantly at 28 days p.i. compared to 21 days p.i. In addition, significantly increased anti-OMV IgA responses were measured in sera (Figure 11H) as well as in lung lysates (Figure 11I) 28 days p.i.

In conclusion, intranasal infection leads to a broad spectrum of systemic antibody subclasses against outer membrane proteins of *B. pertussis* within 14 days p.i., but responses against purified virulent antigens Ptx, Prn, FHA and Fim2/3 remain undetectable until 28 days p.i. Moreover, *B. pertussis*-specific anti-OMV IgA antibodies were secreted in the lungs.

### CD4^+^ T-cell responses after *B. pertussis* infection

**Figure 12:**
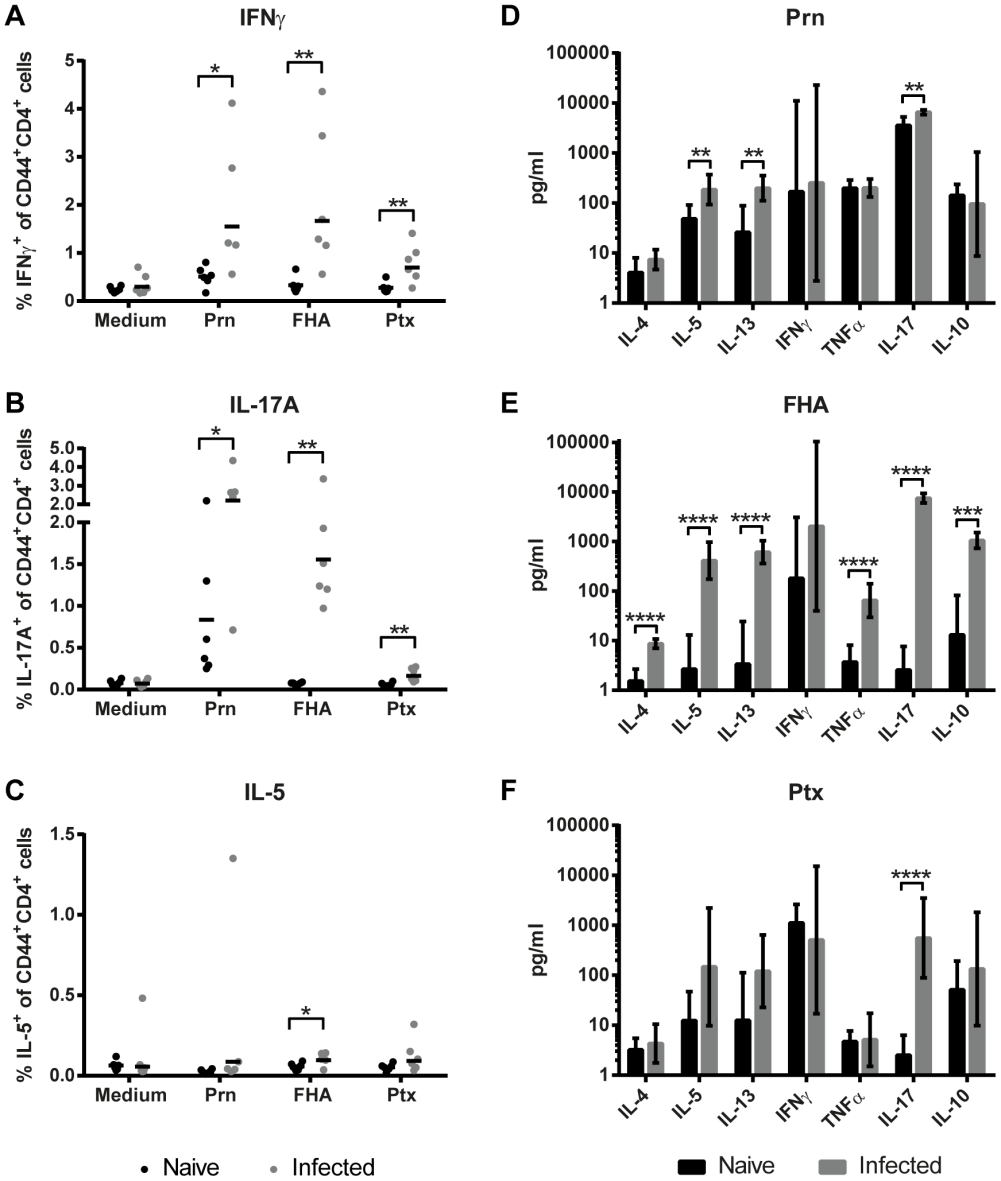
Systemic memory Th-cytokine profiles. (A–C) Splenocytes of naive and infected mice were collected 66 days p.i. and in vitro restimulated with Prn, FHA or Ptx for 8 days. The percentages of IFNγ-, IL-17A-, and IL-5-producing CD3^+^CD4^+^CD44^+^ T-cells were determined using ICS. (D–F) Cytokine levels in supernatant after 7 days of stimulation were determined by using a MIA. Results (mean of n = 6) are corrected for the background level in the presence of medium as control. Statistical differences between the groups were detected for the ICS with a non-parametric Mann-Whitney test and for the MIA with a Student t-test on the log-transformed data. ** = *p*<0.01, *** = *p*<0.001, **** = *p*<0.0001.

Cytokine profiles of antigen-specific memory CD4^+^ T-cells in the spleen were determined 66 days p.i. Splenocytes of naive and infected mice were stimulated for 8 days with Ptx, FHA or Prn, important virulence factors present in the acellular pertussis vaccine. Cytokine profiling on single cell level was established by using intracellular cytokine straining (ICS), while cytokine concentrations in splenic culture supernatants were determined by using a MIA. CD44 expression was used to focus on activated CD4^+^ T-cells. Results of ICS indicate a significant increase in IFNγ- and IL-17A-producing Prn-, FHA-, and Ptx-specific CD4^+^CD44^+^ T-cells after infection (Figure 12A and B). Except for FHA-stimulation, no evidence was found for specific IL-5-producing CD4^+^CD44^+^ T-cells (Figure 12C). Cytokine profiles in culture supernatant revealed a significant increase in TNFα production by splenocytes of infected mice after FHA stimulation and IL-17A production after Prn, Ptx, or FHA stimulation (Figure 12D–F). No changes were observed in IFNγ production in culture supernatants (Figure 12D–F). Notably, FHA stimulation induced besides Th1 and Th17 cytokines an increase in IL-5, IL-13, IL-4, and IL-10 production by splenocytes of infected mice (Figure 12E). IL-5 and IL-13 production occurred also after Prn stimulation (Figure 12D). This might indicate that these three *B. pertussis* antigens induce a mixed Th-subset CD4^+^ T-cell response, of which Th1 and Th17 are the major components. While ICS specifically focuses on CD4^+^CD44^+^ T-cells, multiple cell types can produce the accumulated cytokines present in the splenic culture supernatants after stimulation. The production of IL-17A is exclusive for Th17 cells [69]. Besides Th1 cells, IFNγ could also be produced by CTLs and NK-cells. The high levels of IFNγ produced by splenocytes of naive mice after Ptx stimulation may be explained by TLR4 activation on innate cells by Ptx [70]. A high background of Prn-responding IL-17A^+^CD4^+^CD44^+^ T-cells and IL-17A in supernatant was seen in two out of six naive mice, indicating Prn-activated IL-17A production in unprimed splenocytes.

In summary, CD4^+^ memory T-cell analysis revealed that infection with *B. pertussis* primarily generates specific Th1 and Th17 responses, regarded essential in protection against a *B. pertussis* infection [12], [20], [21].

## Discussion

**Figure 13:**
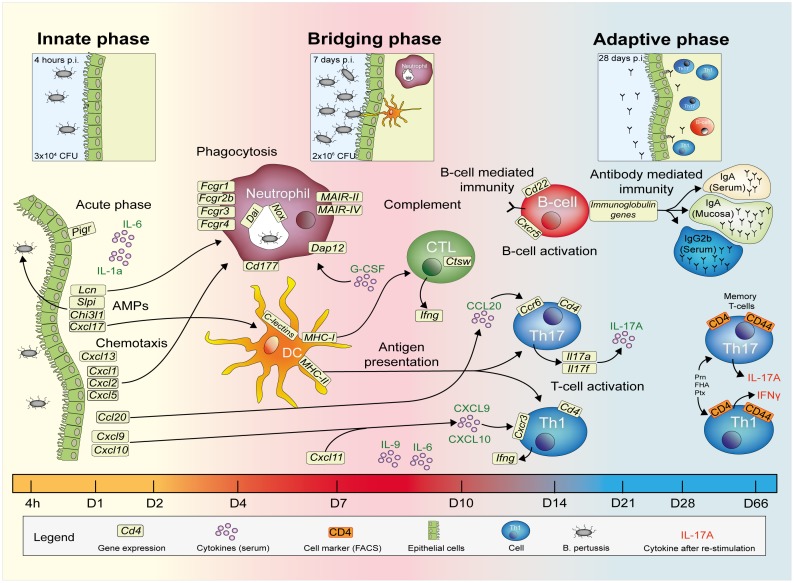
Overview of immune response evolving after *B. pertussis* infection. Merging data from a wide array of functional analyses reveals a comprehensive overview of the immune response of mice upon *B. pertussis* infection. (i) Innate phase, (ii) bridging phase and (iii) adaptive phase are indicated in the response facing variable levels of *B. pertussis* present in the initial inoculum, the exponential growth phase, and in the clearance phase, respectively. Highlights of the study are given, based on lung clearance, relative gene expression levels (lungs), cytokine profiles (serum), cellular response (spleen) and pertussis-specific responses in serum and spleen as depicted in the figure legend.

An integrated systems biology approach was applied to investigate the evolving immune response of mice following a primary intranasal *B. pertussis* infection, considered capable of inducing sterilizing immunity. Data sets from individual analytical platforms, such as microarray, flow cytometry, multiplex immunoassays, and colony counting, addressing different biological samples taken at multiple time points during the immune response were merged. Altogether, a comprehensive overview of immunological processes occurring upon primary infection of mice with *B. pertussis* was constructed in three consecutive phases, as shown in Figure 13: (i) the innate phase, (ii) the bridging phase, and (iii) the adaptive phase.

Highlights featured in this study are: (i) a protracted acute phase response, as part of the innate phase, lasting for at least 28 days, which co-occurred with prolonged antigen exposure; (ii) the recognition of several *B. pertussis* ‘PAMPs’ (pathogen associated molecular pattern), i.e. LPS, DNA and GalNac, (iii) early signatures for recruitment of adaptive immune cells e.g. the cytokine CCL20 that attracts CCR6^+^Th17 cells, (iv) expression of specific membrane markers (e.g. DAP12) and transcription factors that determine the strength and direction of the adaptive immune response. Furthermore, features of adaptive immunity consisted of: (v) IgA secretion in the lungs; (vi) a broad systemic antibody response; (vii) a Th1 and Th17-mediated response in the lungs and (viii) systemic formation of specific memory Th1 and Th17 cells.

Various signatures, associated with the induced protective immune response as presented in Figure 13, are discussed in detail hereafter. The innate phase was characterized by the presence of AMPs, acute phase proteins and chemotactic cytokines as detected by microarray analysis and MIA (Figure 5–7). Expression of the involved genes is most likely initiated by activation of TLR2 and TLR4 by LPS of *B. pertussis* [8], [10]. Up-regulated gene expression of four AMPs (*Chi3l1*, *Cxcl17*, *Lcn2*, *Spli*) was found in the lungs during the whole course of infection. These AMPs contribute to innate protection as was shown for other respiratory pathogens [38]–[40], [42]. In addition, these AMPs have the capacity to recruit immune cells, such as neutrophils, DCs, and monocytes [37], [41] and are therefore important for the induction of subsequent immune responses [36].

Furthermore, the expression of particular cytokines on gene and protein level in the innate phase (Figure 5 and 7), in combination with increased gene expression of cytokine receptors and the change in cellular composition of the spleen (Figure 5 and 8A), gave detailed insight into the immunological processes during the course of infection, as depicted in Figure 13. For example, the gene expression levels of the cytokines *Cxcl1*, *Cxcl2*, *Cxcl5* and *Cxcl17* was in agreement with the recruitment of neutrophils and DCs towards the site of infection [41], [71], [72]. Furthermore, the gene expression of the cytokines *Cxcl9*, *Cxcl10*, *Cxcl11* and *Ccl20* was increased early after infection. CXCL9, CXCL10 and CXCL11 are known to be involved in the recruitment of CXCR3^+^ Th1 cells, and CCL20 in the recruitment of CCR6^+^ Th17 cells [32], [33], [60]. The up-regulation of *Ccr6* and *Cxcr3* genes was observed at day 14 p.i. in the lung (Figure 13).

Remarkably, gene expression of cytokines, acute phase proteins and AMPs was temporary suppressed at 2 days p.i. Simultaneously, the proliferation of bacteria started in the lungs. These results indicate temporary suppression of the immune system by *B. pertussis*. Several immune evasion strategies of *B. pertussis* have been described in literature [73]–[77]. The evasion is attributed to virulence factors, such as adenylate cyclase toxin (ACT), pertussis toxin (Ptx), tracheal cytotoxin (TCT) and filamentous hemagglutinin (FHA). These investigators demonstrated enhanced production of IL-10 and regulatory T-cells, delay of neutrophil recruitment, and inhibition of early chemokine production in mice in relation to secretion of *B. pertussis* virulence factors. In the present study, these immunosuppressive properties of *B. pertussis* might explain the suppression of the differential gene expression observed 2 days p.i. In addition, secretion of OMVs occurs during bacterial colonization [78]. These OMVs are thought to serve as decoy for the host immune response and their immunomodulatory properties support survival of the bacteria [79], [80].

During the bridging phase, the number of bacteria in the lungs increased (Figure 1). The highest number of bacteria in the lungs was found at 7 days p.i. followed by a decrease, suggesting that day 7 is the turning point in the progress of the infection. Simultaneously, microarray analysis suggests the presence of neutrophils, macrophages, and DCs in the lungs based on BioGPS analysis (Figure S1). These cells are likely responsible for (i) phagocytosis, (ii) pathogen recognition, (iii) antigen presentation and (iv) differentiation by expression of cell receptors in the period between 4–10 days p.i. (Figure 13).

The innate immune cells appeared to cause the initial decrease in numbers of bacteria in the lungs from 7 days p.i., because a strong antibody response (IgM, IgG, IgA) (Figure 11) and CD4^+^ cell response were not yet present. Based on gene expression of neutrophil markers, the neutrophil invasion in the lungs probably occurred from 4 days p.i., which is observed on cellular level by McGuirk *et al.* [81]. This influx of cells is most likely orchestrated by IL-17A and G-CSF [66], [67], [69]. In the present study, both cytokines were enhanced in the sera of infected mice. Furthermore, genes involved in phagocytosis were found to be up-regulated (Figure 6) which is important for protection against *B. pertussis* [55], [82].

This study demonstrated also that *B. pertussis* infection activates different pattern recognition receptor (PRR) pathways, such as TLR4, TLR9, DAI and several CLRs (Figure 6). Bacterial ligands, e.g. LPS, DNA and GalNAc trigger these signaling pathways [8], [45], [47]. Up-regulation of genes involved in TLR4-signalling (*Md-1*, *Irf5*) is probably caused by detection of bacterial LPS [8], [10], [43]. Bacterial DNA recognition occurred through the TLR9 pathway (*Ctsk, Irf7*) and the cytosolic DNA sensor (*Dai*). The receptor DAI recognizes both host DNA, upon tissue damage, and bacterial DNA, which leads to an innate immune response [45]. Furthermore, increased gene expression of the CLRs, *Signr7*, *Signr8* and *Mgl1* was observed, which are predominantly expressed on DCs [28], [83]. The enhanced expression of mouse DC-SIGN-related genes *Signr7* and *Signr8* during the *B. pertussis* infection is a novel finding. Despite indications of sensing of 6-sulfo-sialyl Lewis(x) oligosaccharide by Signr7, the exact ligands and role of these receptors remains to be discovered [46]. Recognition of GalNAc via Mgl1 has recently been associated with mast cell activation by *B. pertussis* [47]. An important conclusion is that ligands of *B. pertussis* activate multiple pathogen recognition pathways during the infection. This may be important for the induction of a broad adaptive immune response, including Th1 as well as Th17 cells, mucosal IgA and systemic IgG.

During the bridging phase, up-regulation of MHC class I and II genes were measured, enabling antigen presentation to both CD4^+^ Th-cells and cytotoxic CD8^+^ T-cells (CTLs). Several studies focused on the role of Th-cells because of their important contribution to protect against *B. pertussis* [12], [20], [21]. Nevertheless, the CTLs seem also important for protection against *B. pertussis*, indicated by CTL infiltration in lungs and formation of FHA-specific IFNã-producing CTLs upon *B. pertussis* infection as was previously found in mice and humans [81], [84].

In addition, the bridging phase was characterized by simultaneous gene expression of several receptors on innate immune cells in the lungs and spleen. Interestingly, this included MAIRs, TREMs, semaphorins and DAP12, which is the first evidence that these receptors might play a role in *B. pertussis* infection. Especially DAP12, which is abundantly expressed in lungs, interacts with other membrane receptors and influences cytokine expression [85]. Coupled to MAIR-II, DAP12 is an inhibitor of B-cell responses [86]. Together with TREM2, DAP12 down-regulates TLR and FcR expression [87]. Overall, these former receptors function as an important bridge between innate and adaptive immune responses, determining the strength and direction of the adaptive immune response by the successive activation of lymphocytes, such as CTLs, Th-cells, and B-cells [29], [88]–[91]. Since the exact role of these receptors in the protection against *B. pertussis* remains unresolved, additional research is needed to find whether targeting of these receptors might be a viable strategy to elicit desired immune responses. In summary, the bridging phase suggests the presence of APCs and myeloid cells, such as DCs and neutrophils based on gene expression of membrane markers. However, this was not validated by histological data.

Finally, the adaptive phase was recognized by the formation of T-cell and B-cell responses and a broad humoral response (Figure 13). In this same period, the naive mice were able to clear *B. pertussis* from their lungs. Gene expression, cytokine secretion and cellular analysis revealed presence of activated CD4^+^ Th-cells. The highest number of CD4^+^ Th-cells was detected at 14 days p.i., which is in agreement with a study showing the largest influx of Th17 cells in the lungs after *B. pertussis* infection at 14 days p.i. [12]. Phenotyping of CD4^+^ Th-cells, based on cytokine profiles in serum (protein) and lung (genes) and expression of specific transcription factors or membrane markers, suggests a broad Th-response in the present study. The Th1 response in the lungs was characterized by *Ifng* and *Stat1* expression [92]. Gene expression of the mucosal homing receptor *Ccr6*, *Card11*, *Ccl19*, *Ccl21a*, *Ccl21c*, and signature cytokines *Il17a* and *Il17f* might indicate Th17 cells in the lungs [32], [69], [93]–[96]. Upon re-stimulation of splenocytes with purified *B. pertussis* antigens, Prn- and FHA-specific Th1 and Th17 type CD4^+^ T-cells could be detected in the memory phase (66 days p.i.) (Figure 12). In addition, enhanced IL-9 levels in serum suggest activation of Th9 cells and Th17 cells as these cells produce large amounts of IL-9 [64], [65]. However, Tregs, NKT-cells and mast cells are also sources of IL-9. In summary, the different phases of T-cell development were mapped; the expansion phase of naive T-cells in the spleen (2 days p.i.), the effector phase in the lungs (14 days p.i.), contraction phase in the spleen (21 days p.i.) and finally the specific memory T-cells (66 days p.i.) (Figure 5, 8A, 10 and 12). Data from this study suggests generation of local specific Th1/Th17 cells in the lungs. Th1 cells, which produce IFNγ are important for clearance of *B. pertussis*. Postponed clearance was shown in IFNã-depleted mice [97]. Th17 cells have been indicated as key cells to control bacterial infections at mucosal sites [98]. Recent studies revealed that Th17 cells increase the clearance of *B. pertussis* after an intranasal infection in animals [12], [13], [99].

Infection with *B. pertussis* induces also B-cell and antibody mediated immunity. Microarray analysis showed increased gene expression of the BCR in the lungs. In addition, gene expression of IgM, IgG and IgA was observed in lungs and spleen. Whereas, most genes for antibody isotypes were found up-regulated in both tissues, IgM was down-regulated in the spleen (Figure 6 and 10). This was confirmed by the detection of IgA and IgG (IgG1, IgG2a and IgG2b) after 14 days p.i. However, IgM antibodies were not found systemically (Figure 11). In mice, IgG1 is associated with a Th2-like response, while IgG2a (in Balb/c mice) and IgG2b suggest induction of a Th1 response [100]. IgG2b is also linked to Th17 lymphocytes [101]. All antibody responses in the present study were directed against whole cell *B. pertussis* and outer membrane vesicles, but not against the *B. pertussis* antigens that are typically present in acellular vaccines: Ptx, Prn, FHA and Fim2/3. However, memory Th1/Th17 cells were detected upon re-stimulation with Prn and FHA (Figure 12). The polymeric immunoglobulin receptor (pIgR) was highly expressed during the whole course of *B. pertussis* infection in the lungs. pIgR is essential for the transport of IgA into the mucus and is seen as a bridge between innate and adaptive mucosal responses [102], [103]. The expression of pIgR was drastically enhanced 14 days p.i., leading to secretion of mucosal IgA in the lungs (Figure 5 and 11). Interestingly, the increased pIgR expression coincided with IL-17A production in the lungs and sera (Figure 5 and 7), which is in agreement with the finding that Th17-mediated responses influence the local humoral response by inducing pIgR expression and elevating secretory IgA levels [104]. Therefore, the transport of IgA to the mucosa, which is orchestrated by IL-17A via the induction of pIgR, supports an important role for local immunity. In summary, *B. pertussis* infection induces a broad humoral response, which was observed by gene and protein expression.

Our study also contains several conceptual points for discussion. First, altered gene expression in the spleen caused by *B. pertussis* infection was modest, except for 21 days p.i. Two possible reasons can be given: (i) the infection initiated a local immune response in the lungs and (ii) gene expression is required to be rather strong to exceed the significance threshold. After the lungs and the draining lymph nodes, the spleen is the third organ in line involved in the generation of the immune response. Therefore, gene expression effects could be less abundant. Furthermore, the spleen consists of many cell types with different gene expression profiles, so changes in expression levels in a single cell type might not have been detected. Cell sorting of individual cell types and subsequent gene expression profiling could overcome this problem. Second, specific antibody and T-cell responses were only measured for a limited set of available purified antigens. The inclusion of other antigens, such as outer membrane proteins, would perhaps have allowed a more detailed analysis. Third, in this study we propose murine infection-induced immune signatures as a benchmark to be used in the development of improved human pertussis vaccines. Since *B. pertussis* is not a natural pathogen for mice, translation of the results obtained in this study to the human situation remains to be interpreted with caution. Nevertheless, using a (naive) murine model has several advantages. It is regarded an important small animal model for pertussis vaccine development [105] and enables to dissect the local and systemic immune response in great detail, as shown in this study, rather than using blood or nasal washes from humans undergoing a *B. pertussis* infection. Furthermore, the role of individual gene signatures and products could be assessed in future research in mice by using knockin or knockout strains, or by functional interventions. Encouraging is that many observations made in this study, such as the rapid clearance upon reinfection and involvement of Th17 and Th1 responses, are in line with recent studies on *B. pertussis* infection in baboons, which resemble the human situation more closely [13], [15]. Translation of biomarkers from mouse to man however should be performed with care.

Overall, our study describes the molecular and cellular sequence of events that eventually lead to the induction of protective immunity to pertussis, including local immunity in the lungs, which is not induced by current vaccines. Especially the inductions of Th1 and Th17 cells and IgA in the lungs are regarded key elements in superior immunity. Our data can be used to select vaccine concepts that resemble infection, with respect to e.g. administration route and prolonged antigen exposure, and immune signatures elicited. Local delivery of vaccines may be an important advancement for improved protection as was previously demonstrated with a live attenuated pertussis vaccine [106]. Also adjuvants could be selected and optimized that can steer host immunity of inactivated vaccines towards an infection-like immune response with minimal adverse effects. The molecular and cellular fingerprint of the local immune response discovered in *B. pertussis* infected mice thus can provide a strong guidance for developing improved pertussis vaccines.

## Methods

### Ethics Statement

The independent ethical committee for animal experimentations ‘Dierexperiment Commissie (DEC)’ of the National Institute for Public Health and the Environment (RIVM) reviewed the animal experiments in this study according to the guidelines provided by the Dutch Animal Protection Act. The committee approved documents with the identification numbers ‘DPA201100230’ and ‘DPA201100348’.

### Materials

*Bordetella pertussis* strain B1917 [107] was isolated in The Netherlands in 2000 and was kindly provided by Frits Mooi. Bordet Gengou agar plates with 15% sheep blood were purchased from BD (Cat. No. 254400, BD, The Netherlands). Verweij medium was obtained from Bilthoven Biologicals (BBio, Bilthoven, The Netherlands). PBS (pH 7.2) and Roswell Park Memorial Institute Media 1640 (RPMI) were purchased from Invitrogen (Gibco, Invitrogen). All RPMI media used in this experiment were supplemented with 10% fetal calf serum (FCS, Hyclone, New Zealand), 100 units penicillin, 100 units streptomycin, and 2.92 mg/ml L-glutamine (p/s/g; Invitrogen). Shockbuffer for splenocyte isolation consisted of 8.3 g/L NH_4_CL, 1 g/L NaHCO_3_ and 5000 IE/L Heparine dissolved in dH_2_O (pH 7.4; ice cold) and was filtered (0.22 µm) before use. FACS buffer consisted of PBS pH 7.2 supplemented with 0.5% BSA (A3803, Sigma Aldrich, Germany) and 0.5 mM EDTA (ICN Biomedicals). Horseradish peroxidase (HRP) labelled goat-anti-mouse IgG and *R*-Phycoerythrin (RPE)-conjugated goat-anti-mouse IgM, IgA, IgG, IgG1, IgG2a, IgG2b and IgG3 were obtained from Southern Biotech (Southern Biotech, United States) Fluorescently labeled antibodies for flow cytometry, anti-CD3-Pacific blue (17A2), anti-CD4-Pacific blue (GK1.5), anti-IA-IE-PerCP-Cy5.5 (M5/114.15.2), anti-33D1-APC (33D1) and anti-IL-5-APC were purchased from Biolegend (ITK, The Netherlands). Fluorescently labeled antibodies, anti-CD4-APC (RM4-5), anti-CD8a-PerCP (53 6.7), anti-CD69-FITC (H1.2 F3), anti-Gr-1-PE (RB6-8C5), anti-CD19-FITC (1D3), anti-CD14-PE (rmC5-3), anti-DX5-APC (DX5), anti-CD11b-PE (M1/70), anti-CD40-FITC (HM40-3), anti-CD44-FITC (IM7) and anti-IFNγ-PE were purchased from BD (BD Biosciences, The Netherlands). Fluorescently labeled antibodies, CD11c – PE-Cy7 (N418) and anti-IL-17a-PerCP-Cy5.5 were obtained from eBioscience (eBioscience, Austria). Live/dead staining was purchased from Invitrogen (LIVE/DEAD Fixable Aqua Dead Cell Stain Kit; Invitrogen). Anti-CD16/CD32 was purchased from BD (BD, The Netherlands).

### Cultivation of *Bordetella pertussis*

*Bordetella pertussis* strain B1917 was grown on Bordet-Gengou agar plates for 4 days at 35°C. Subsequently, multiple colonies were transferred onto new plates and grown for 24 hours at 35°C, which was repeated 3 times. Colonies on each plate were harvested in 1.5 ml Verweij medium [108] and the suspensions were pooled. After centrifugation (30 min., 1932 g), cells were washed with 15 ml Verweij medium. Subsequently, bacterial suspensions of approximately 2×10^10^ cfu/ml were resuspended in Verweij medium containing 15% glycerol (v/v) and aliquots of 0.25 ml were flash frozen in ethanol and dry ice and stored at −80°C.

### Challenge culture

A stock of *B. pertussis* B1917 was diluted with Verweij medium to a final concentration of 5×10^6^ cfu/ml. The number of cfu was confirmed by plating 100 µL of the infection suspension (1∶2000 in Verweij medium) on Bordet-Gengou agar plates. Plates were incubated for 4 days at 35°C and cfu were counted by using a colony counter (ProtoCOL, Synbiosis, Cambridge, United Kingdom).

### Animal experiment

Female BALB/c mice (Harlan, The Netherlands), 8-weeks-old, were divided in groups of three animals and housed in cages (macrolon III including filter top). Three healthy mice were euthanized at day 0 and considered as naive group. Other mice were intranasally infected under anesthesia (isoflurane/oxygen), with 2×10^5^ cfu *B. pertussis* B1917 in 40 µl Verweij medium at day 0. Groups of animals (n = 3) were euthanized after 2, 4, 6 hours, and after 1, 2, 4, 7, 10, 14, 21 and 28 days of infection, respectively. At 56 days p.i. five groups of primary infected animals and five groups of naive animals (n = 3) were infected as previously described and euthanized after 4 hours, and after 2, 7, 10 and 14 days of infection. In addition, six infected and six healthy mice were euthanized at day 21 and day 66 for the analysis of CD4^+^ T-cell responses. Mice were bled, under anesthesia (isoflurane/oxygen), by orbital bleeding and sacrificed by cervical dislocation.

Whole blood from each mouse was collected in a blood collection tube (MiniCollect 0.8 ml Z Serum Sep GOLD, Greiner Bio-One, Austria). After coagulation (10 min. at room temperature), sera were taken after centrifugation (10 min., 3000 g) and stored at −80°C. Lung lobes were excised. The right lobe was placed in 900 µl Verweij medium and kept at room temperature. The left lobe was placed in 1 ml RNAlater (Qiagen), incubated overnight at 4°C and stored at −80°C. The spleen was excised and divided in two equal parts. One piece was placed in 5 ml of RPMI medium and kept on ice. The other piece was placed in 1 ml RNAlater (Qiagen), incubated overnight at 4°C and stored at −80°C. For the analysis of CD4^+^ T-cell responses, whole spleen was collected and placed in 5 ml of RPMI medium and kept on ice.

### Colonization assay

Lung tissue in 900 µl Verweij medium was homogenized for 15 seconds using a Labgen 7 Homogenizer (Cole Palmer, Schiedam, The Netherlands). Homogenizer was sanitized between samples with ethanol (80%) and water. Lung homogenates were serially diluted (undiluted, 1∶10, 1∶100 or 1∶1000) in Verweij medium depending on the expected number of cfu. Suspensions of 100 µl were plated on Bordet-Gengou agar plates and incubated for 4 days at 35°C. The numbers of colonies were counted by using a colony counter (ProtoCOL, Synbiosis, Cambridge, United Kingdom) and calculated as cfu per mouse.

### RNA isolation

Lung and spleen tissue was homogenized by using a Labgen 7 Homogenizer (Cole Palmer, Schiedam, The Netherlands) in 700 and 2100 µl Qiazol (Qiagen Benelux, Venlo, The Netherlands), respectively. RNA isolation was performed using a miRNeasy Mini Kit with DNAse treatment (Qiagen Benelux, Venlo, The Netherlands) according to the manufacturer's protocol. RNA concentrations were determined by UV spectroscopy (Tech3 module, Synergy Mx, BioTek, Winooski, United States). RNA quality was determined by using electrophoresis (RNA nano 6000 kit, 2100 Bioanalyzer, Agilent Technologies, Amstelveen, The Netherlands). Results were given as RNA integrity numbers (RIN) between 1 nd 10 (manual Bioanalyzer). Samples with a RIN of 7 or higher were used for microarray analysis. For microarray analysis of lung tissue, RNA concentrates of individual mice were analyzed for the following time points: naive, 4 hours, 2 days, 4 days, 7 days, 10 days, 14 days, 21 days and 28 days post infection (p.i.). For spleen tissue, samples of individual mice were analyzed for the following time points: naive, 4 hours, 1 day, 2 days, 4 days, 7 days, 10 days, 14 days, 21 days and 28 days p.i.

### Microarray analysis

Amplification, labeling and hybridization of RNA samples to microarray chips was carried out at the Microarray Department of the University of Amsterdam, The Netherlands, as described previously [109].

Briefly, 500 ng total RNA of each sample was amplified (aRNA) according to the Agilent QuickAmp kit manual (Agilent technologies). A common reference sample for either lung or spleen tissue was made by pooling equimolar amounts of aRNA from individual samples of the respective tissues. Cy3 and Cy5 mono-reactive dyes (GE Healthcare) were used to label individual samples and the common reference sample, respectively. Labeling was performed with 10 ml of CyDye solution and incubated for 1 hour before the reaction was quenched by adding 5 ml 4 M hydroxylamine (Sigma-Aldrich). Purification was performed by using a clean-up kit (E.Z.N.A. MicroElute RNA Clean Up Kit, Omega Bio-Tek, Norcross, United States). The yields of amplified RNA and incorporation of CyDye were determined by using a spectrophotometer (NanoDrop ND-1000, NanoDrop products, Wilmington, United States).

Hybridization mixture was prepared by adding a dried mixture (1∶1) of sample (Cy3) and common reference (Cy5) together with sample tracking control (STC, Roche NimbleGen) and hybridization cocktail (NimbleGen Arrays User's Guide –Gene Expression Arrays Version 5.0, Roche NimbleGen). Samples were incubated successively for 5 min. at 95°C and 5 min. at 42°C. Hybridization was performed by loading a sample onto a microarray (NimbleGen 12×135 k Mus musculus, Roche, Germany) containing probes for 44,170 genes with 3 spots per target probe. Hybridization was performed with a NimbleGen Hybridization System 4 (Roche NimbleGen) for 20 hours at 42°C. After washing (NimbleGen Arrays User's Guide – Gene Expression Arrays Version 5.0), slides were scanned in an ozone-free room with a microarray scanner (Agilent DNA microarray scanner G2565CA, Agilent Technologies). Each microarray corresponded to labeled RNA from specific tissue (lung or spleen) from one individual mouse. Feature extraction was performed with NimbleScan v2.5 (Roche NimbleGen) resulting in a table containing individual probe signal intensities for both dyes. Complete raw and normalized microarray data and their MIAME compliant metadata from this publication have been submitted to the GEO database (www.ncbi.nlm.nih.gov/geo) and assigned the identifier GSE53294.

### Data analysis of gene expression

Quality control was performed on raw data by means of Cy3-Cy5 scatter plots, and by comparing signal average and distribution across slides. All slides passed quality control. Raw microarray data for gene-coding probes were normalized in R (www.r-project.org), by using a four step approach [109]: (1) natural log-transformation, (2) quantile normalization of all scans, (3) correcting the sample spot signal for the corresponding reference spot signal and (4) averaging data from replicate probe spots. Further analysis of normalized data of 44170 probes was performed in R and Microsoft Excel.

Genes differentially expressed between experimental groups (naive and various time points p.i.) were identified by using ANOVA. Fold ratio induction or repression of individual genes was calculated by comparing mean gene expression levels of infected groups to the group with naive mice. Data are presented as the average normalized gene expression levels of three mice per group. Probes were considered differentially expressed if they met the following two criteria: (i) a p-value<0.001 (ANOVA), which corresponds to a Benjamini-Hochberg False discovery rate (FDR) [110] of <5%; and (ii) an absolute fold ratio >1.5 (infected compared to naive mice) for at least one time point. If multiple probes corresponding to the same gene were significant, their data were averaged to remove redundancy for further analysis. Gene expression of each group was compared with the group of naive mice. Differences in gene expression were visualized in heat maps, (GeneMaths XT, Applied Maths, St-Martens-Latem, Belgium). Genes were arranged according to similar expression patterns in time at which genes exceeded the fold ratio cut-off of 1.5. To facilitate visual interpretation of heat maps, only induction (red) and repression (green) of gene expression levels with fold ratios >1.5 are visualized, therefore presenting fold ratios <1.5 as naive level (black).

Functional enrichment with an over-representation analysis (ORA) was carried out by using DAVID [111] based on Gene Ontology Biological Processes (GO-BP) and Kyoto Encyclopedia of Genes and Genomes (KEGG). Cell-specific or tissue-specific gene sets were obtained by using data from BioGPS [112], [113]. For each cell type or tissue, gene expression data were compared to the average of all tissues included in the data set. The top 100 genes, which had the highest tissue-specific expression and were included on the NimbleGen array, were selected. If the BioGPS dataset included multiple entries for the same cell type (e.g., CD4^+^ and CD8^+^ T-cells), the resulting sets were combined in our subsequent analysis.

### Whole cell *B. pertussis* ELISA

Immunoglobulin G responses against *B. pertussis* were determined in sera by an ELISA. Therefore, *B. pertussis* B1917 was heat-inactivated for 45 min. at 56°C, after which ELISA plates (Immunolon 2HB, Thermo Scientific) were coated overnight at room temperature with 100 µl/well whole cell *B. pertussis* (OD_590 nm_ = 0.1 in PBS). Plates were washed with 0.03% (v/v) Tween 80 in water. Then, plates were incubated for 1 hour at 37°C with 100 µl/well serial dilutions of serum samples in PBS, pH 7.2 (Gibco) with 0.1% (v/v) Tween 80. Plates were washed twice and incubated (1 hour, 37°C) with 100 µl/well of Goat anti Mouse IgG-HRP (1∶5000) in PBS with 0.1% (v/v) Tween 80 and 0.5% (w/v) Protifar (Nutricia). After washing twice, 100 µl/well peroxidase substrate (4.2 mM Tetra Methyl Benzidine and 0.012% H_2_O_2_ in 0.11 M sodium acetate buffer, pH 5.5.) was added, plates were incubated at room temperature and the reaction terminated after 10 min. by adding 100 µl/well, 2 M H_2_SO_4_ (BBio, Bilthoven, The Netherlands). Finally, the absorbance was recorded at 450 nm with a plate reader (BioTek reader EL808, Bio-Tek, USA) and antibodies against whole cell *B. pertussis* are presented in OD450 signal, because a reference is not available.

### Multiplex immunoassay (MIA) for antibody response

Responses of IgM, IgA, total IgG and the IgG subclasses (IgG1, IgG2a, IgG2b and IgG3) against *B. pertussis* antigens P.69 pertactin (P.69 Prn), filamentous hemagglutinin (FHA), pertussis toxin (Ptx), combined fimbria type 2 and 3 antigens (Fim2/3) and outer membrane vesicles B1917 (OMV B1917) were determined in sera by using a MIA. Outer membrane vesicles from *B. pertussis* B1917 were produced as previously described [114] with additional changes. Conjugation of antigens and OMVs to beads was performed as described previously [115]. Serum was diluted 1/100 in PBS containing 0.1% Tween 20 and 3% bovine serum albumin and mixed 1∶1 with conjugated beads. After incubation with *R*-Phycoerythrin (RPE)-conjugated anti-mouse IgM (1∶100), IgA (1∶100), IgG (1∶200), IgG1 (1∶200), IgG2a (1∶40), IgG2b (1∶200) and IgG3 (1∶200), samples were analyzed by using a Bio-Plex system (Bio-Plex 200, BioRad). Total IgG titers (U/ml) against *B. pertussis* antigens were calculated from mean fluorescent intensity (MFI) by using a reference from the National Institute for Biological Standards and Control (Code 99/520, NIBSC, England) in the Bio-Plex Manager software (Bio-Rad Laboratories). Results for IgM, IgG, IgG subclasses and IgA antibodies against OMV B1917 are presented in fluorescent intensity (F.I.), because a reference is not available.

### Lung lysate preparation and IgA antibody assay

The lung lysates used for the colonization assay were centrifuged (2000 g, 10 min.) and filtered (0.22 µm Millex-GV, Merck KGaA, Darmstadt, Germany) to remove cell debris and bacteria. Filtrates were stored at −80°C. Prior to analysis, lung lysates were diluted 1∶10 in PBS containing 0.1% Tween 20 and 3% bovine serum albumin. Analysis of IgA antibodies in the lung lysate was performed against *B. pertussis* antigens (Ptx, Prn, FHA, and Fim2/3) and OMV B1917 as described in the section multiplex immunoassay (MIA) for antibody response.

### Splenocyte isolation

Spleen tissue was forced through a 70 µm cell strainer (BD Falcon, BD Biosciences, USA) and washed two times with 5 ml medium (RPMI, FCS, p/s/g) to obtain a single cell suspension. After centrifugation (550 g, 5 min.), 1 ml shock buffer was added to cell pellet to lyse erythrocytes. After one minute, shock incubation was stopped by adding 25 ml medium. For cell count (CASEY model TT, Innovatis, Roche, Germany), cells were centrifuged and resuspended in 1 ml medium.

### Cellular composition of spleen

Splenocytes (1×10^6^) were transfered to each well of a V-bottom 96-wells plate. After centrifugation (400 g, 3 min.), pellets were washed with 100 µl FACS buffer. Fc-specific receptors on cells were blocked with anti-CD16/CD32 in FACS buffer (2%, v/v) by 5 min. incubation on ice to prevent aspecific binding of labeled antibodies to cells. Cells were stained after centrifugation by using a mixture (100 µl) of labeled antibodies (30 min., 4°C). To determine the number and identity of cells, three different panels of labeled antibodies were used to prevent overlapping fluorescence spectra. Panels consisted of 1) total T-cell population (CD3), CD4 T-cell (CD4), CD8 T-cell (CD8), early activated T-cell (CD69), and neutrophil (Gr-1); 2) T-cell (CD3), B-cell (CD19), monocyte (CD14), and natural killer cell (DX5); 3) T-cell (CD3), macrophage (CD11b), DC (33D1, CD11c), MHC class II (IA/IE), and activation marker (CD40). Data acquisition was performed with a FACSCanto II (BD Biosciences, USA) and data analysis was done by using FlowJo software (Tree Star Inc., USA). Debris and dead cells were excluded by using forward/side scatter characteristics and live/dead staining, respectively.

### Stimulation of splenocytes

Splenocytes were resuspended in the culture medium IMDM (Gibco) +8% FCS+p/s/g +20 µM β-mercaptoethanol (Sigma) and cultured (24-wells plate; 6×10^6^ cells/well) for 7 days at 37°C in a humidified atmosphere containing 5% CO_2_. Culturing was in the presence of either the culture medium, or the culture medium supplemented with Prn P.69 (in house expressed and purified as described previously [116]), heat-inactivated (15 min at 95°C) Ptx (Kaketsuken, Japan) or FHA (Kaketsuken, Japan) at a final concentration of 1 µg/ml. Supernatants were collected after 7 days for cytokine analysis. Subsequently, cells were transferred to U-bottom 96-wells plates (5×10^5^ cells/well) and stimulated overnight by using the same antigens and conditions and used for intracellular cytokine staining.

### Intracellular cytokine staining

The intracellular cytokine staining was performed on 8-days stimulated splenocytes by using a Cytofix/Cytoperm Fixation/Permeabilization Solution Kit (BD Biosciences, USA). Briefly, cells were incubated during the last 5 hours of stimulation with 10 µg/ml Golgiplug (BD Biosciences), 1 µg/ml αCD28 (BD Pharmingen), and 1 µg/ml αCD49d (BD Pharmingen). Cells were stained after washing with FACS buffer with anti-CD4-Pacific blue, anti-CD44-FITC and live/dead staining. Subsequently, the cells were fixed, permeabilized, and stained with anti-IFNγ-PE, anti-IL-5-APC and anti-IL-17a-PerCP-Cy5.5. Data from fluorescence-activated cells were acquired on FACS Canto II (BD Biosciences) and analyzed with FlowJo software (Tree Star Inc., USA). CD44 was used to distinguish between naive and antigen experienced T-cells. IL-5 was used as a Th2 cytokine, IFNγ as a Th1 cytokine, and IL-17 as a Th17 cytokine.

### Multiplex immunoassay (MIA) for cytokines

Cytokine (IL-4, IL-5, IL-10, IL-13, IL-17A, TNFα, and IFNγ) concentrations (pg/ml) were determined in culture supernatants of 7 days stimulated splenocytes of individual mice by using a MIA (Milliplex mouse cytokine 7-plex luminex kit; Merck KGaA, Darmstadt, Germany). Cytokine (Eotaxin, G-CSF, GM-CSF, IFN-γ, IL-10, IL-12 (p40), IL-12 (p70), IL-13, IL-15, IL-17A, IL-1α, IL-1β, IL-2, IL-3, IL-4, IL-5, IL-6, IL-7, IL-9, IP-10, KC, LIF, LIX, M-CSF, MCP-1, MIG, MIP-1α, MIP-1β, MIP-2, RANTES, TNF-α, VEGF) concentrations (pg/ml) present in serum were determined by using a MIA (Milliplex MAP Mouse Cytokine/Chemokine - Premixed 32 Plex; Merck KGaA, Darmstadt, Germany) according to the manufacturer's instructions. Briefly, serum/supernatant (25 µl) was diluted in supplied assay buffer (1/1; v/v), mixed with 25 µl conjugated beads and incubated overnight at 4°C on a plate shaker. Subsequently, beads were washed twice with washing buffer by using a Bio-Plex handheld magnetic washer (BioRad, USA) and incubated with 25 µl detection antibodies (1 hour, 20–25°C) followed by incubation with 25 µl streptavidin-phycoerythrin (30 min., 20–25°C). After washing twice with 200 µl washing buffer, beads were resuspended in 150 µl PBS. Samples were analyzed using a multiplex system (Bio-Plex 200, BioRad, USA). In total, 50 beads per region were analyzed in 100 µl sample volume. Mean fluorescence intensity (MFI) of each cytokine was converted into pg/ml by using a dilution series of a standard (3.2–10,000 pg/ml) and Bio-Plex Manager software 5.0 (Bio-Rad, USA).

### MIP-3α (CCL20) cytokine ELISA

Serum concentration of Macrophage Inflammatory Protein 3α (MIP3α/CCL20) was determined by ELISA (Mouse MIP-3α ELISA kit, RAB0061, Sigma-Aldrich, Germany) according to the manufacturer's manual. In short, serum (50 µl) was diluted in assay buffer A (1/1; v/v) and incubated in an antibody-coated ELISA plate (overnight, 4°C, gentle shaking). After washing four times with 300 µl wash buffer, wells were incubated with 100 µl biotinylated antibody (1 hour, room temperature, gentle shaking). After washing four times with 300 µl wash buffer, wells were incubated with 100 µl streptavidin solution (45 min., room temperature, gentle shaking). After washing four times, wells were incubated with 100 ml TMB one-step substrate reagent (30 min., RT) and reaction stopped with 50 ml stop solution. Finally, the absorbance was recorded at 450 nm with a plate reader (BioTek reader EL808, Bio-Tek) and converted to MIP-3α concentration by using a dilution series of a standard (2.06–1,500 pg/ml) and Gentech 5 software (BioTek).

### Statistical analysis

Cellular composition of spleen, antibody titers and cytokines levels were analyzed by using a one-way ANOVA with multiple comparisons of all time points post infection vs. naive mice followed by a Dunnett t-test. ICS data was analyzed by using a Mann-Whitney t-test. For cytokine profiling after re-stimulation of splenocytes, MIA data was first log-transformed before t-test analysis. In all corresponding figures, *p*-values are represented as * = *p*<0.05, ** = *p* <0.01, *** = *p*<0.001 and **** = *p*<0.0001.

## Supporting Information

Figure S1 **Cell type comparison analysis for lung gene expression.** Data represent gene profiles in the lung per cell type or tissue extracted from BioGPS. Results from B-cells, mast cells, T-cells, dendritic cells, neutrophils and lung are depicted. (Mean of n = 3).
(TIF)

Figure S2 **Cellular composition of the spleen as function of time after** ***B. pertussis*** **infection.** The percentage of (A) CD14^+^ cells (CD14^+^), (B) NK-cells (DX5^+^), (C) macrophages (CD11b^+^), (D) neutrophils (Gr-1^+^), (E) T-cells (CD3^+^, CD4^+^CD8^−^ and CD8^+^CD4^−^), (F) B-cells (CD19^+^) and (G) dendritic cells (33D1^+^) in splenocytes were analyzed over time. *p*-values were determined by one-way ANOVA with multiple comparison compared to naive mice (* = *p*<0.05, ** = *p*<0.01, *** = *p*<0.001 and **** = *p*<0.0001). (mean of n = 3). An increased percentage of CD14^+^ cells (monocytes, DCs and macrophages) was found 1 day p.i., followed by natural killer (NK) cells and macrophages at 2 days p.i. There was a gradual increase in the percentage of neutrophils in the spleen until 7 days p.i. Furthermore, an increased percentage of T-cells, distinguished by the CD3 marker, was found 2 and 4 days p.i. A similar increase was observed for both CD4^+^CD8^−^ and CD8^+^CD4^−^ T-cell subsets, indicating that this increase was not specific for either T-helper or cytotoxic T-cells. The CD4^+^ cells increased significantly 21 days p.i. and remained constant until at least 28 days p.i. B-cells decreased during two periods: 2–4 and 10–14 days p.i. Finally, a significant increase in percentage of DCs (33D1^+^) was detected 7 days p.i.
(TIF)

Figure S3 **Flow cytometric-gating strategy for CD14+, Gr-1+, CD19+ and CD11b+ cell population analysis in splenocytes.** Each FACS plot shows results from one mouse of the group. Per cell type, the change in numbers of cells is visible over time for CD14+, Gr-1+, CD19+ and CD11b+ cell populations. Time points 2 hours, 6 hours and 10 days p.i. were excluded in this figure. (*) FACS plot for CD14+ cells at day 4 and day 7 p.i. were manually adapted by BiExponential transformation in FlowJo for better visualization without influencing the gating.
(TIF)

Figure S4 **Flow cytometric-gating strategy for CD3+, CD4+, CD8+, DX5+ and 33D1+ cell population analysis in splenocytes.** Each FACS plot shows results from one mouse of the group. Per cell type, the change in numbers of cells is visible over time for CD3+, CD4+, CD8+, DX5+ and 33D1+ cell populations. Time points 2 hours, 6 hours and 10 days p.i. were excluded in this figure. (*) FACS plot for 33D1+ cells at day 1, day 7 and day 14 p.i. were manually adapted by BiExponential transformation in FlowJo for better visualization without influencing the gating.
(TIF)

Figure S5 **Splenic gene expression profiles of cell types and tissues extracted from BioGPS databases.** Data represent gene profiles of the following cell types or tissues: B-cells, neutrophils, mast cells, T-cells, macrophages, dendritic cells (DCs), lung, lymph nodes and spleen. (Mean of n = 3).
(TIF)

Figure S6 **Genes differentially regulated in both lung and spleen.** Comparison of transcriptomic data in lung and spleen revealed an overlap of 72 genes differentially regulated in both tissues. Genes divided in 4 groups based on expression pattern. Functional annotation showed that genes in group 2 were mostly involved in immunological processes. (Mean of n = 3).
(TIF)

Figure S7 **IgG antibody response in serum against Ptx, Prn, FHA and Fim2/3 after** ***B. pertussis*** **infection.** IgG antibody titers against four purified *B. pertussis* antigens; (A) pertussis toxin (Ptx), (B) pertactin (Prn), (C) filamentous hemagglutinin (FHA), and (D) Fimbriae 2 and 3 (Fim2/3) were determined using a multiplex immunoassay (mean of n = 3). The antibody titers were determined in mouse sera 14, 21 and 28 days after an intranasal infection with *B. pertussis*. No antibodies were detected for Ptx, Prn and Fim2/3. For FHA, 1 out of 3 mice showed induced titers antibody titers at 21 days p.i.
(TIF)

Table S1 **Full list of genes expressed in the lungs.** This file contains the full list of significantly expressed genes obtained by microarray analysis of the lungs, which can be used for additional in-depth information for each gene. In total 558 genes were significantly altered compared to naïve mice. The expression pattern for each gene is given including the fold changes at each time point. In addition to the official gene symbol (obtained from the Nimblegen array), the full gene name, synonyms and gene ID are given. Synonyms are not given for all genes but only for genes that were included in detail in the manuscript. Gene ID is a linked to gene information on the NCBI website. Subsequently, there are three columns with gene information obtained in databases; (i) BioGPS, (ii) Gene Ontology – Biological Processes (GO-BP), (iii) KEGG pathways. Pull-down menus can be used to search for specific information in the full gene list. For BioGPS, information about cell and tissue specific gene expression is given for the following databases; B-cells (B), dendritic cells (DC), lung (Lu), lymph node (LN), mast cells (MC), macrophages (MF), neutrophils (N), spleen (Sp) and T-cells (T). For GO-BP the following biological processes were included; ‘acute inflammatory response’, ‘acute-phase response’, ‘antigen processing and presentation’, ‘cell activation’, ‘chemotaxis’, ‘defense response’, ‘immune response’, ‘inflammatory response’, ‘intracellular signaling cascade’, ‘phagocytosis’, ‘regulation of apoptosis’, ‘response to bacterium’ and ‘transcription’. For KEGG the following pathways were included; ‘Antigen processing and presentation’, ‘Apoptosis’, ‘B cell receptor signaling pathway’, ‘Cell adhesion molecules (CAMs)’, ‘Chemokine signaling pathway’, ‘Complement and coagulation cascades’, ‘Cytokine-cytokine receptor interaction’, ‘Endocytosis’, ‘Leukocyte transendothelial migration’, ‘MAPK signaling pathway’, ‘NOD-like receptor signaling pathway’ and ‘Toll-like receptor signaling pathway’.
(XLSX)

Table S2 **Full list of genes expressed in the spleen.** This file contains the full list of significantly expressed genes obtained by microarray analysis of the spleen, which can be used for additional in-depth information for each gene. In total 798 genes were significantly altered compared to naïve mice. The expression pattern for each gene is given including the fold changes at each time point. In addition to the official gene symbol (obtained from the Nimblegen array), the full gene name, synonyms and gene ID are given. Synonyms are not given for all genes but only for genes that were included in detail in the manuscript. Gene ID is a linked to gene information on the NCBI website. Subsequently, there are three columns with gene information obtained in databases; (i) BioGPS, (ii) Gene Ontology – Biological Processes (GO-BP), (iii) KEGG pathways. Pull-down menus can be used to search for specific information in the full gene list. For BioGPS, information about cell and tissue specific gene expression is given for the following databases; B-cells (B), dendritic cells (DC), lung (Lu), lymph node (LN), mast cells (MC), macrophages (MF), neutrophils (N), spleen (Sp) and T-cells (T). For GO-BP the following biological processes were included; ‘acute inflammatory response’, ‘antigen processing and presentation’, ‘cell activation’, ‘chemotaxis’, ‘chromatin assembly’, ‘defense response’, ‘hemopoiesis’, ‘immune response’, ‘inflammatory response’, ‘intracellular signaling cascade’, ‘regulation of apoptosis’, ‘response to bacterium’ and ‘transcription’. For KEGG the following pathways were included; ‘Antigen processing and presentation’, ‘Apoptosis’, ‘B cell receptor signaling pathway’, ‘Cell adhesion molecules (CAMs)’, ‘Chemokine signaling pathway’, ‘Complement and coagulation cascades’, ‘Cytokine-cytokine receptor interaction’, ‘Endocytosis’, ‘Leukocyte transendothelial migration’, ‘MAPK signaling pathway’, ‘NOD-like receptor signaling pathway’ and ‘Toll-like receptor signaling pathway’.
(XLSX)
