# Supplementary figures and images for: Molecular Signatures of the Evolving Immune Response in Mice following a Bordetella pertussis Infection
Source: PLoS One. 2014 Aug 19;9(8):e104548. doi: 10.1371/journal.pone.0104548 (PMC4138111; doi:10.1371/journal.pone.0104548)

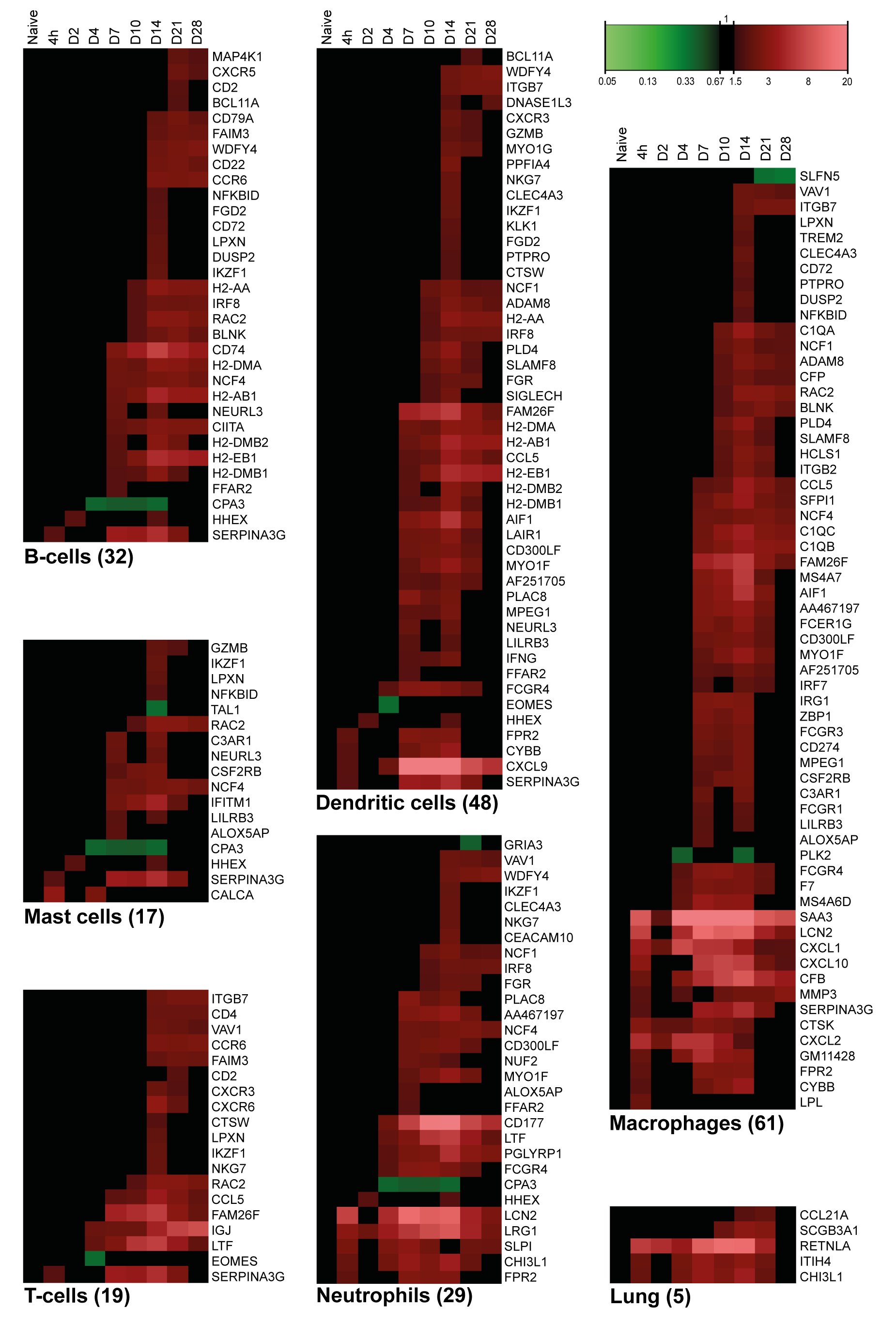

Supplement: Figure S1 — Cell type comparison analysis for lung gene expression. Data represent gene profiles in the lung per cell type or tissue extracted from BioGPS. Results from B-cells, mast cells, T-cells, dendritic cells, neutrophils and lung are depicted. (Mean of n = 3). (TIF) [file pone.0104548.s001.tif]

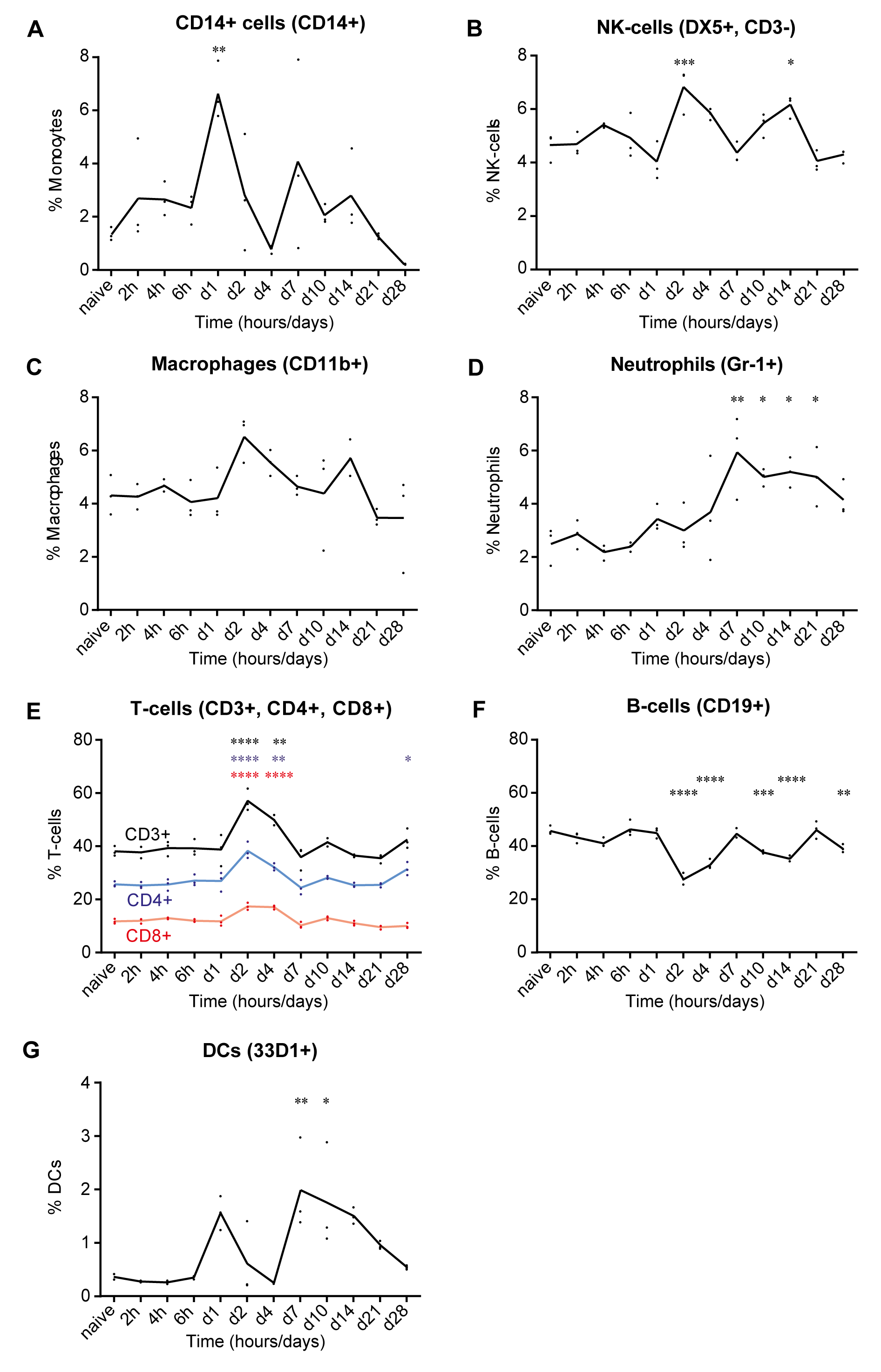

Supplement: Figure S2 — Cellular composition of the spleen as function of time after B. pertussis infection. The percentage of (A) CD14+ cells (CD14+), (B) NK-cells (DX5+), (C) macrophages (CD11b+), (D) neutrophils (Gr-1+), (E) T-cells (CD3+, CD4+CD8− and CD8+CD4−), (F) B-cells (CD19+) and (G) dendritic cells (33D1+) in splenocytes were analyzed over time. p-values were determined by one-way ANOVA with multiple comparison compared to naive mice (* = p<0.05, ** = p<0.01, *** = p<0.001 and **** = p<0.0001). (mean of n = 3). An increased percentage of CD14+ cells (monocytes, DCs and macrophages) was found 1 day p.i., followed by natural killer (NK) cells and macrophages at 2 days p.i. There was a gradual increase in the percentage of neutrophils in the spleen until 7 days p.i. Furthermore, an increased percentage of T-cells, distinguished by the CD3 marker, was found 2 and 4 days p.i. A similar increase was observed for both CD4+CD8− and CD8+CD4− T-cell subsets, indicating that this increase was not specific for either T-helper or cytotoxic T-cells. The CD4+ cells increased significantly 21 days p.i. and remained constant until at least 28 days p.i. B-cells decreased during two periods: 2–4 and 10–14 days p.i. Finally, a significant increase in percentage of DCs (33D1+) was detected 7 days p.i. (TIF) [file pone.0104548.s002.tif]

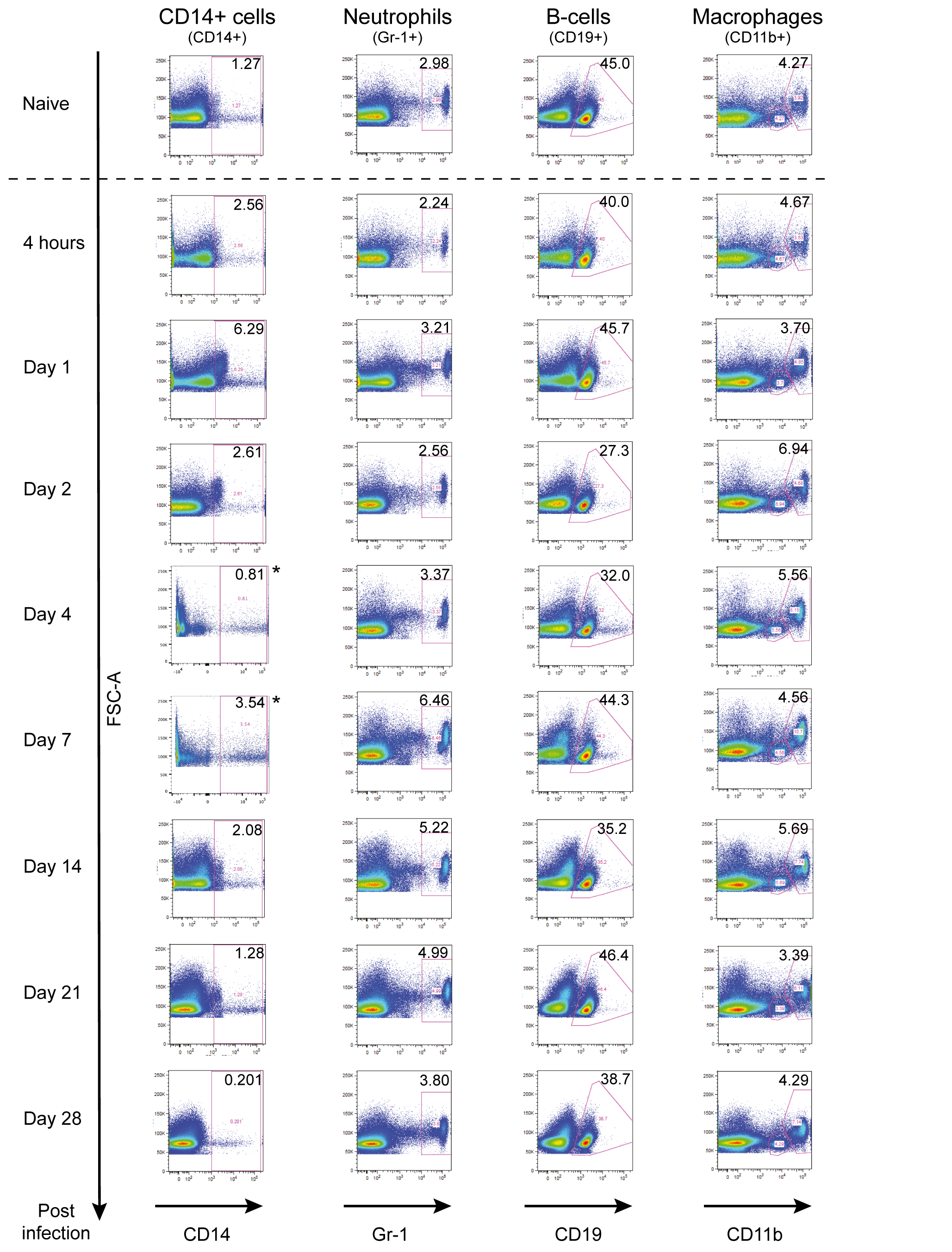

Supplement: Figure S3 — Flow cytometric-gating strategy for CD14+, Gr-1+, CD19+ and CD11b+ cell population analysis in splenocytes. Each FACS plot shows results from one mouse of the group. Per cell type, the change in numbers of cells is visible over time for CD14+, Gr-1+, CD19+ and CD11b+ cell populations. Time points 2 hours, 6 hours and 10 days p.i. were excluded in this figure. (*) FACS plot for CD14+ cells at day 4 and day 7 p.i. were manually adapted by BiExponential transformation in FlowJo for better visualization without influencing the gating. (TIF) [file pone.0104548.s003.tif]

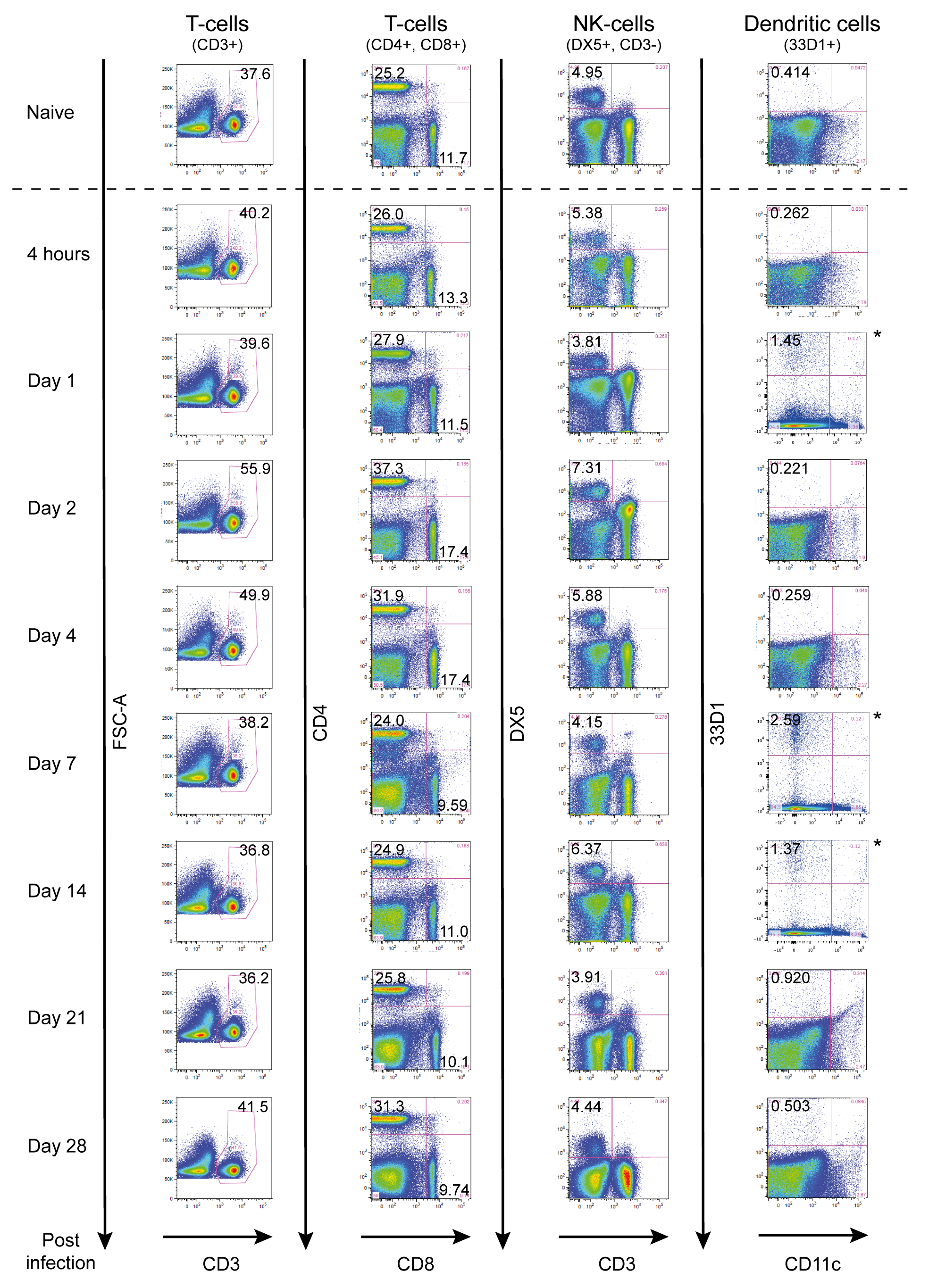

Supplement: Figure S4 — Flow cytometric-gating strategy for CD3+, CD4+, CD8+, DX5+ and 33D1+ cell population analysis in splenocytes. Each FACS plot shows results from one mouse of the group. Per cell type, the change in numbers of cells is visible over time for CD3+, CD4+, CD8+, DX5+ and 33D1+ cell populations. Time points 2 hours, 6 hours and 10 days p.i. were excluded in this figure. (*) FACS plot for 33D1+ cells at day 1, day 7 and day 14 p.i. were manually adapted by BiExponential transformation in FlowJo for better visualization without influencing the gating. (TIF) [file pone.0104548.s004.tif]

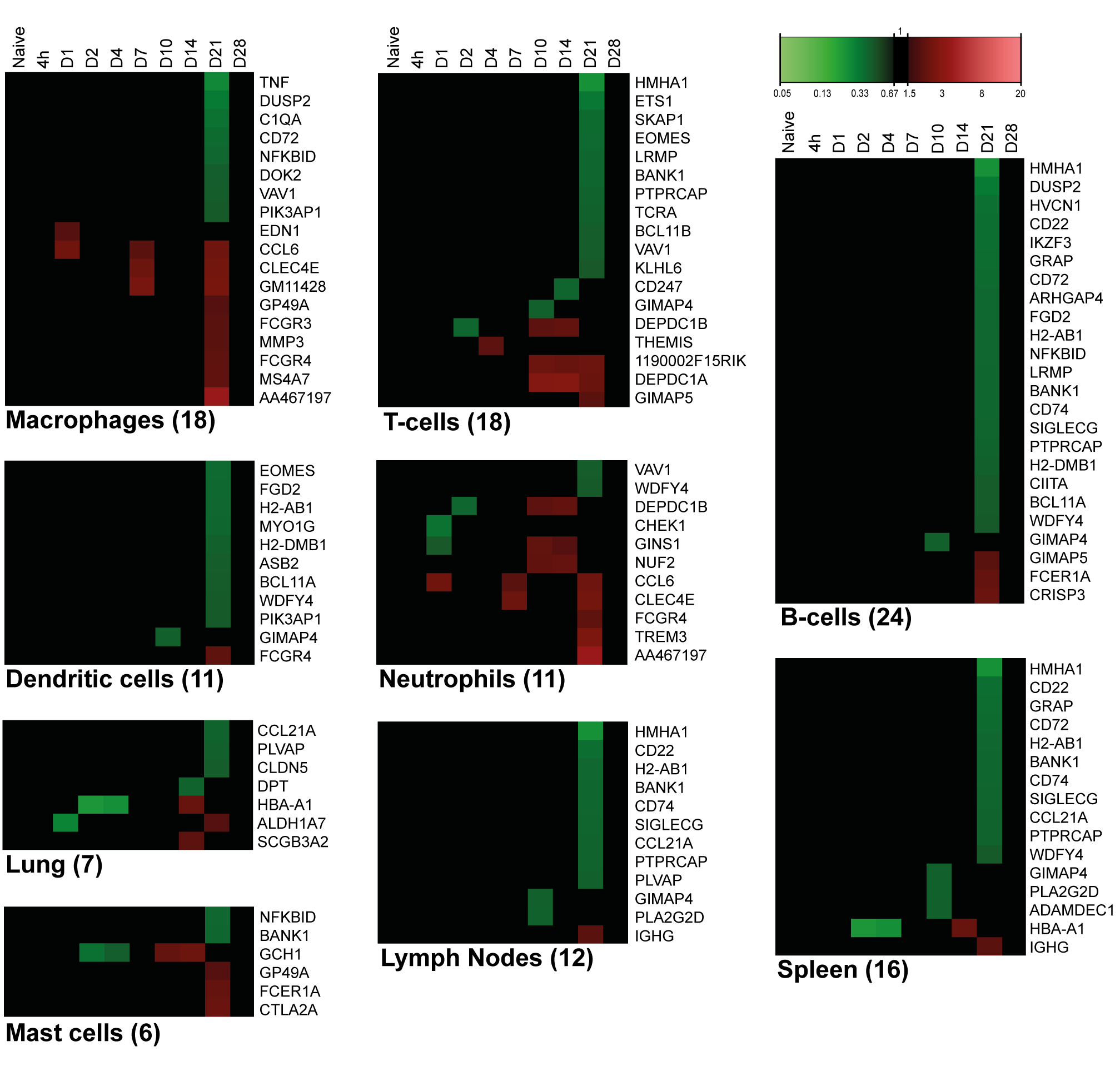

Supplement: Figure S5 — Splenic gene expression profiles of cell types and tissues extracted from BioGPS databases. Data represent gene profiles of the following cell types or tissues: B-cells, neutrophils, mast cells, T-cells, macrophages, dendritic cells (DCs), lung, lymph nodes and spleen. (Mean of n = 3). (TIF) [file pone.0104548.s005.tif]

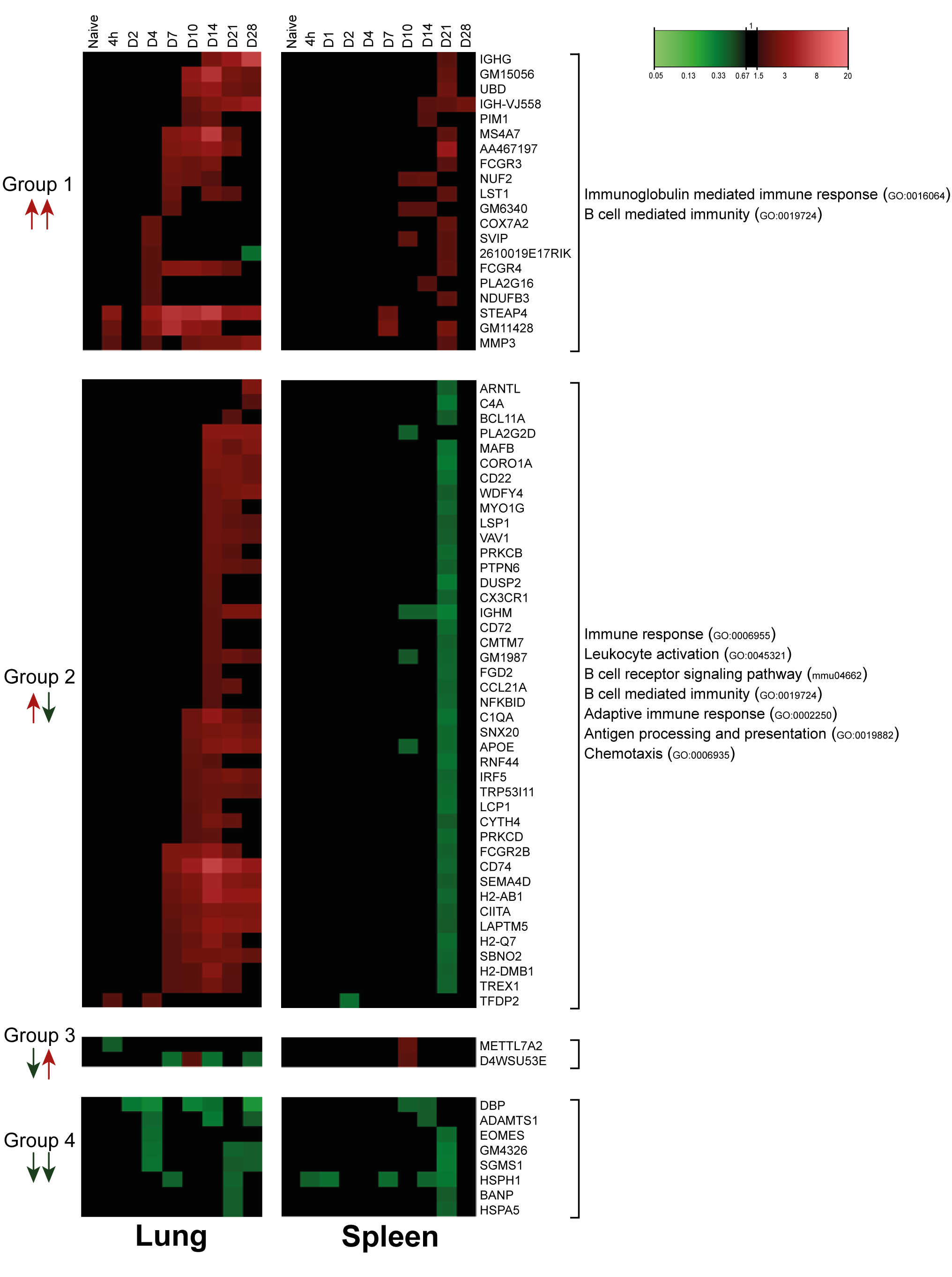

Supplement: Figure S6 — Genes differentially regulated in both lung and spleen. Comparison of transcriptomic data in lung and spleen revealed an overlap of 72 genes differentially regulated in both tissues. Genes divided in 4 groups based on expression pattern. Functional annotation showed that genes in group 2 were mostly involved in immunological processes. (Mean of n = 3). (TIF) [file pone.0104548.s006.tif]

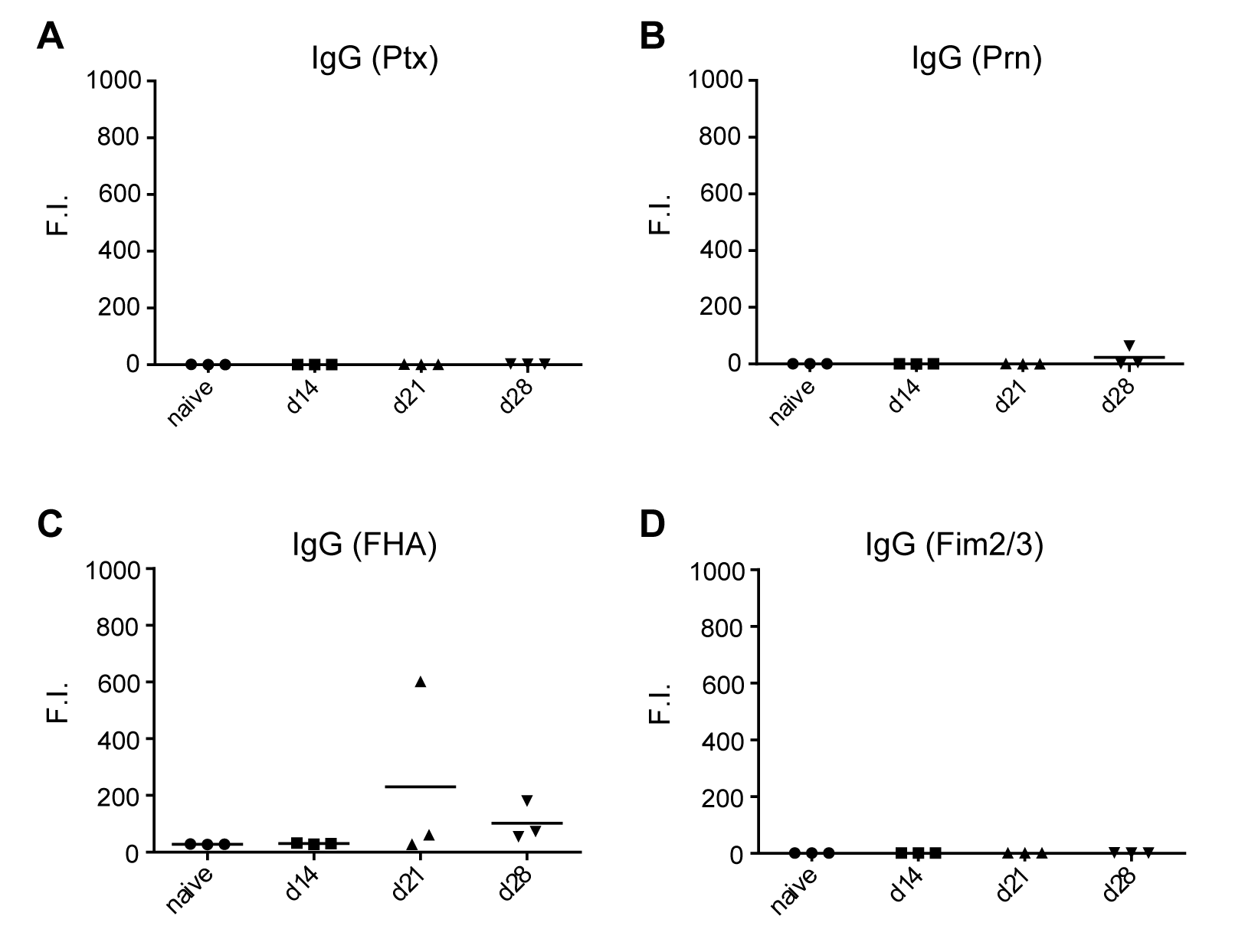

Supplement: Figure S7 — IgG antibody response in serum against Ptx, Prn, FHA and Fim2/3 after B. pertussis infection. IgG antibody titers against four purified B. pertussis antigens; (A) pertussis toxin (Ptx), (B) pertactin (Prn), (C) filamentous hemagglutinin (FHA), and (D) Fimbriae 2 and 3 (Fim2/3) were determined using a multiplex immunoassay (mean of n = 3). The antibody titers were determined in mouse sera 14, 21 and 28 days after an intranasal infection with B. pertussis. No antibodies were detected for Ptx, Prn and Fim2/3. For FHA, 1 out of 3 mice showed induced titers antibody titers at 21 days p.i. (TIF) [file pone.0104548.s007.tif]
